# An intramolecular hydrogen bond-promoted “green” Ugi cascade reaction for the synthesis of 2,5-diketopiperazines with anticancer activity†

**DOI:** 10.1039/d2ra04958a

**Published:** 2022-11-21

**Authors:** Jie Li, Jiu Hong Huang, Jing Ya Wang, Zhi Gang Xu, Zhong Zhu Chen, Jie Lei

**Affiliations:** College of Pharmacy, National & Local Joint Engineering Research Center of Targeted and Innovative Therapeutics, IATTI, Chongqing University of Arts and Sciences Chongqing 402160 China

## Abstract

We report a “green chemistry”-based Ugi cascade reaction to furnish a series of 2,5-diketopiperazines (through nucleophilic attack of amides upon ketones in Ugi adducts) at moderate-to-good yields. Investigation with the MTT assay revealed compound (±) 5c to exhibit potent anticancer activities against acute myeloid leukaemia (MV411; IC_50_ = 1.7 μM) and acute T lymphocyte leukaemia (Jurkat; IC_50_ = 5.7 μM) cell lines.

## Abstract 2

We report an intramolecular hydrogen bond-promoted Ugi cascade reaction for solvent-free synthesis of 2,5-diketopiperazines. Compounds (±) 5c and (±) 5e displayed potent antitumor activity against acute myeloid leukaemia and prostate cancer cell lines, respectively.

## Graphical abstract

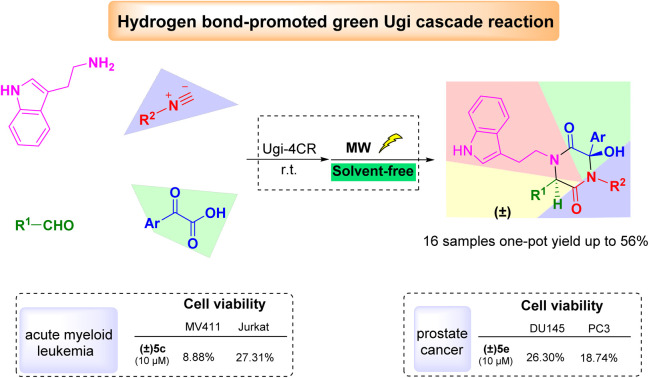

“Green chemistry” concepts can guide advanced syntheses in organic chemistry.^1^ The four-component Ugi reaction (U-4CR) perfectly matches with the concepts of green chemistry. U-4CR employs four commercially available reagents to furnish dipeptide-like products and has been used widely to construct diverse heterocyclic scaffolds.^2^ Among nitrogen derivatives, a cyclic dipeptide (2,5-diketopiperazine) is an important scaffold in medicinal chemistry and drug discovery. In this scaffold, various substituents exhibit numerous medicinally relevant properties, such as the: potent antimalarial activity of Diatretol I^3^ and its relevant scaffold of lepistamides II;^4^ antibacterial activity of thaxtomin A III;^5^ antibiotic activity of bicyclomycin IV^6^ as well as its activity towards microtubule depolymerization V,^7^ 5HT ligands VI^8^ and radical scavenging VII^9^ (Fig. 1).

**Fig. 1:**
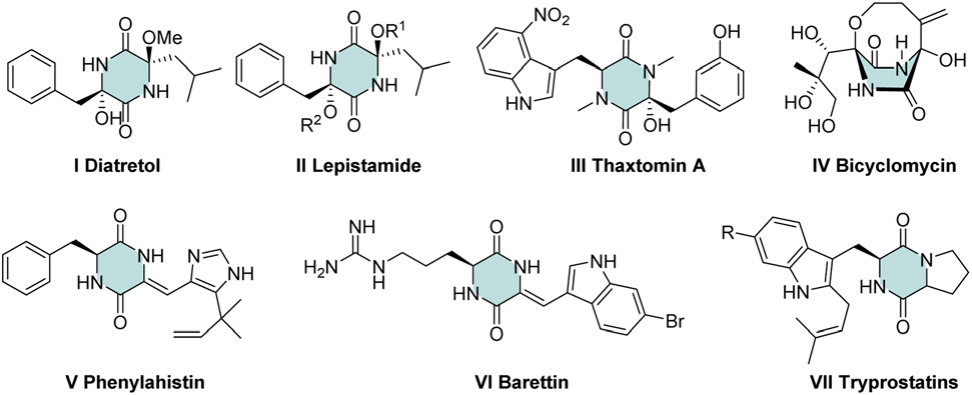
Bioactive derivatives of 2,5-diketopiperazine.

Therefore, 2,5-diketopiperazine has been considered to be a “privileged” scaffold in drug discovery. Efficient strategies for rapid construction of structurally diverse scaffolds have been demanded. Some methods related to this particular cyclic dipeptide have been published. One method involves using functionalized piperazinones as starting reagents to provide a structural elaboration of the piperazine-2,5-dione motif.^10^ Another method involves using 2-aminohydrazide or an amino acid as starting materials for the preparation of diketopiperazines *via* multi-step sequences (Scheme 1a).^11^ Recently, an acid-catalysed-reaction was applied to facilitate monodehydro-2,5-diketopiperazine (DKP), which was limited to *N*-a-ketoacyl amino acid amides (Scheme 1b).^12^ Later on, an acyclic compound was subjected to direct base-mediated keto-amide cyclization to afford DKPs in a 10 : 1 dr, but only one example was reported.^13^ The transformation of amino-acid substrates to 2,5-diketopiperazine was reported by Parac-Vogt and co-workers, which was conducted in the presence of the Zr/Wells-Dawson 2 : 2 complex in dimethyl sulfoxide (Scheme 1c).^14^ Revuelta and co-workers stated that the replacement of a ketone with an aromatic aldehyde as carbonyl compounds cannot be tolerated *via* isocyanide-based multicomponent reactions (iMCRs),^15^ but desired compounds were not detected using Ugi adducts as starting materials under basic conditions. Even though there were several examples in which Ugi adducts could be converted to 2,5-diketopiperazine scaffolds, only a few compounds were synthesized and a narrow substrate scope confined the applicability of synthetic methodologies.

**Scheme 1:**
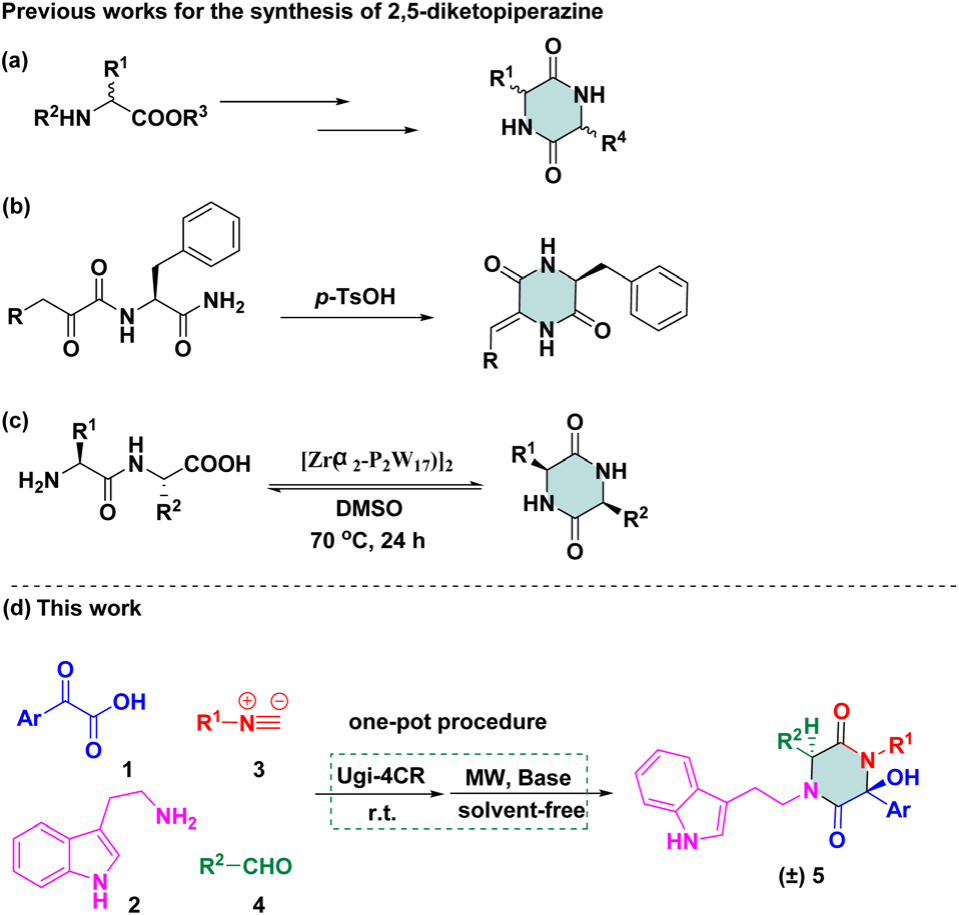
Reported and designed synthetic routes of 2,5-diketopiperazines.

We were interested in the development of new cascade reactions based on iMCRs. Hence, we analysed the sequence mechanism employed to construct pyrrolopyridinones, indoline-piperidinones and 4-imidazolidinones by our research team.^16^ Ugi adducts were formed using a mixture of benzoylformic acid, ethyl glyoxylate, isocyanide and an aromatic amine (containing an electron-withdrawing group). We postulated that, in this Ugi core, the reaction started with nucleophilic attack of a carbanion to afford the four-membered azetidin-2-one ring (Scheme 2, path A).^17^ In contrast, secondary amides would be deprotonated and used as nucleophiles to attack the α-ketone, thereby resulting in a 2,5-diketopiperazine ring. We assumed that replacement of ethyl glyoxylate with an aromatic aldehyde reduced the reactivity of the nucleophilic carbon. In this case, transformation of the four-member ring intermediate was inhibited, which promoted the nucleophilic attack of the amide to access 2,5-diketopiperazines (Scheme 2, path B). We wondered if the two nucleophilic sites (secondary amide and α-carbon) in this unique reaction had a distinct pathway to finish cyclization through a Ugi sequence.^18^ Following this idea, we herein present a base-promoted approach to the synthesis of 2,5-diketopiperazines. This new method broadens the substrate scope of a microwave-assisted one-pot protocol under solvent-free conditions (Scheme 1d). Importantly, the principles of green chemistry and absence of solvents carry several advantages over a traditional organic process: simple operation, less toxic and hazardous chemicals and an accelerated reaction rate.^19^

**Scheme 2:**
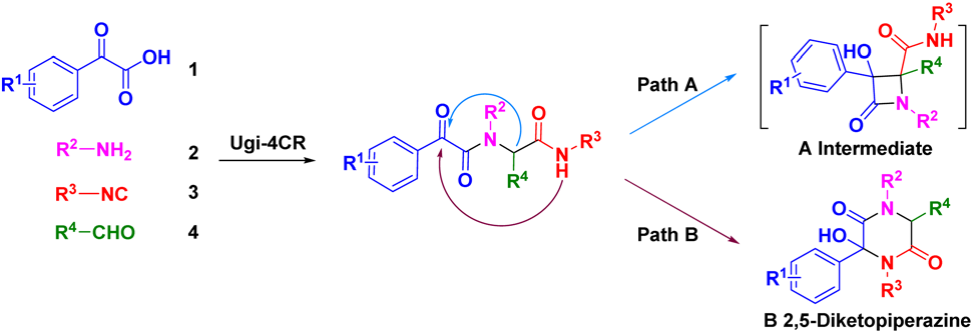
Nucleophilic attack of carbanion (path A) or secondary amide (path B).

To check our hypothesis, a highly functionalized precursor C was constructed readily through the Ugi four-component reaction of benzoylformic acid 1a, tryptamine 2a, benzyl isocyanide 3a and benzaldehyde 4a (Table 1). The protic solvent in the reaction could dissolve the substrates thoroughly, but could also polarize imines to access Ugi products in good yields. Inspired by these findings, we attempted to construct a one-pot procedure to obtain the final products. After the Ugi reaction had been completed, the crude Ugi adduct C was treated under certain reaction conditions. Subsequently, the protic solvent of methanol (MeOH) was selected to evaluate the effect of bases. Screening the organic base and inorganic base revealed that 2.0 equiv. of diisopropanolamine (DIPA) showed promising results and gave the desired product in MeOH with a yield of 31%. To further increase the yield of (±) 5a, we switched our attention to solvent effects, and different solvents were assessed. *n*-BuOH (*n*-butanol) was a powerful solvent under microwave irradiation at 100 °C for 20 min, which provided a much higher yield than MeOH (57%, entry 11). To our delight, the Ugi adduct C was obtained by removing the solvent. Then, 2.0 equiv. of DIPA was added directly to the mixture, followed by microwave irradiation at 100 °C for 20 min. The final product (±) 5a was isolated with 53% yield. In addition, a solvent-free strategy has been implemented widely and attracted considerable attention recently. This is an alternative, sustainable route that could overcome the drawbacks of conventional methods of organic synthesis, such as tedious workup procedures and generation of large amounts of solvent waste. To pursue green chemistry, we increased the reaction temperature from 100 °C to 180 °C, and a satisfactory yield (71%) of the target compound was obtained under microwave irradiation at 160 °C for 20 min (entry 18). The optimized reaction condition was determined to be 2.0 equiv. DIPA without a solvent under microwave irradiation at 160 °C for 20 min.

**Table 1:** Optimization of reaction conditions^a^

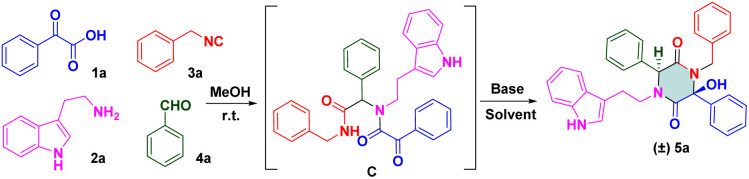					
Entry	Base	Solvent	Temp. (°C)	Time (min)	Yield^b^ (%)
1	DBU	MeOH	MW 100	20	NR
2	TEA	MeOH	MW 100	20	27
3	DABCO	MeOH	MW 100	20	19
4	DIPEA	MeOH	MW 100	20	22
5	DIPA	MeOH	MW 100	20	31
6	NaHCO_3_	MeOH	MW 100	20	NR
7	Na_2_CO_3_	MeOH	MW 100	20	NR
8	NaOH	MeOH	MW 100	20	NR
9	Na^t^OBu	MeOH	MW 100	20	NR
10	DIPA	EtOH	MW 100	20	32
11	DIPA	*n*-BuOH	MW 100	20	45
12	DIPA	*i*-PrOH	MW 100	20	26
13	DIPA	DMF	MW 100	20	NR
14	DIPA	Toluene	MW 100	20	NR
15	DIPA	MeCN	MW 100	20	15
16	DIPA	—	MW 100	20	53
17	DIPA	—	MW 130	20	62
**18**	**DIPA**	**—**	**MW 160**	**20**	**71**
19	DIPA	—	MW 180	20	Complex

a The reaction was carried out with 1a (0.3 mmol), 2a (0.3 mmol), 3a (0.3 mmol) and 4a (0.3 mmol) in MeOH at room temperature. After the solvent had evaporated, the crude product C was treated directly with a base (2.0 equiv.) to afford (±) 5a. DBU = 1,8-diazabicycloundec-7-ene; TEA = trietylamine; DABCO = 1,4-diazabicyclo[2.2.2]octane; DIPEA = *N*,*N*-diisopropylethylamine; DIPA = diisopropanolamine; DMF = dimethylformamide; MW = microwave.

b Isolated yield.

Upon determination of the optimized reaction condition, we evaluated the scope of microwave-promoted Ugi cascades for the synthesis of 2,5-diketopiperazines. This green Ugi cascade worked well and there was no distinct difference in the yield of desired products. The structure of the isolated compound (±) 5a (CCDC 2009028) was confirmed unequivocally by X-ray crystallography (Scheme 3). Various benzaldehydes were used as aldehyde sources to furnish Ugi adducts that were converted efficiently to the corresponding products. Structures (±) 5b–f suggested that aldehydes containing electron-withdrawing groups (F, Br, Cl, NO_2_) provided final yields of compounds from 60% to 70%. Unsurprisingly, aldehydes containing electron-donating groups (*e.g.*, Me, MeO, *t*-Bu) could be tolerated well, and afforded yields from 79% to 83% ((±) 5g–i). These results were consistent with data from our previous work^16,17^ and suggested that the nucleophilic attack occurred between the carbanion and ketone of benzoylformic acid. For the isocyanide moiety, commercially available 4-methoxyl benzyl isocyanide was investigated to broaden the substrate scope and evaluate reaction compatibility. The yields from reactions (±) 5j–m did not decrease dramatically. Crude Ugi adducts with a 4-methoxyl benzoylformic acid moiety and 4-bromo benzoylformic acid moiety were conducted under a standard condition to generate final compounds (±) 5n and (±) 5o with good yields. A substrate containing a heterocycle furan was also processed well; a one-pot isolated yield of 70% was achieved for (±) 5p*via* a post-Ugi cascade sequence under a solvent-free condition.

**Scheme 3:**
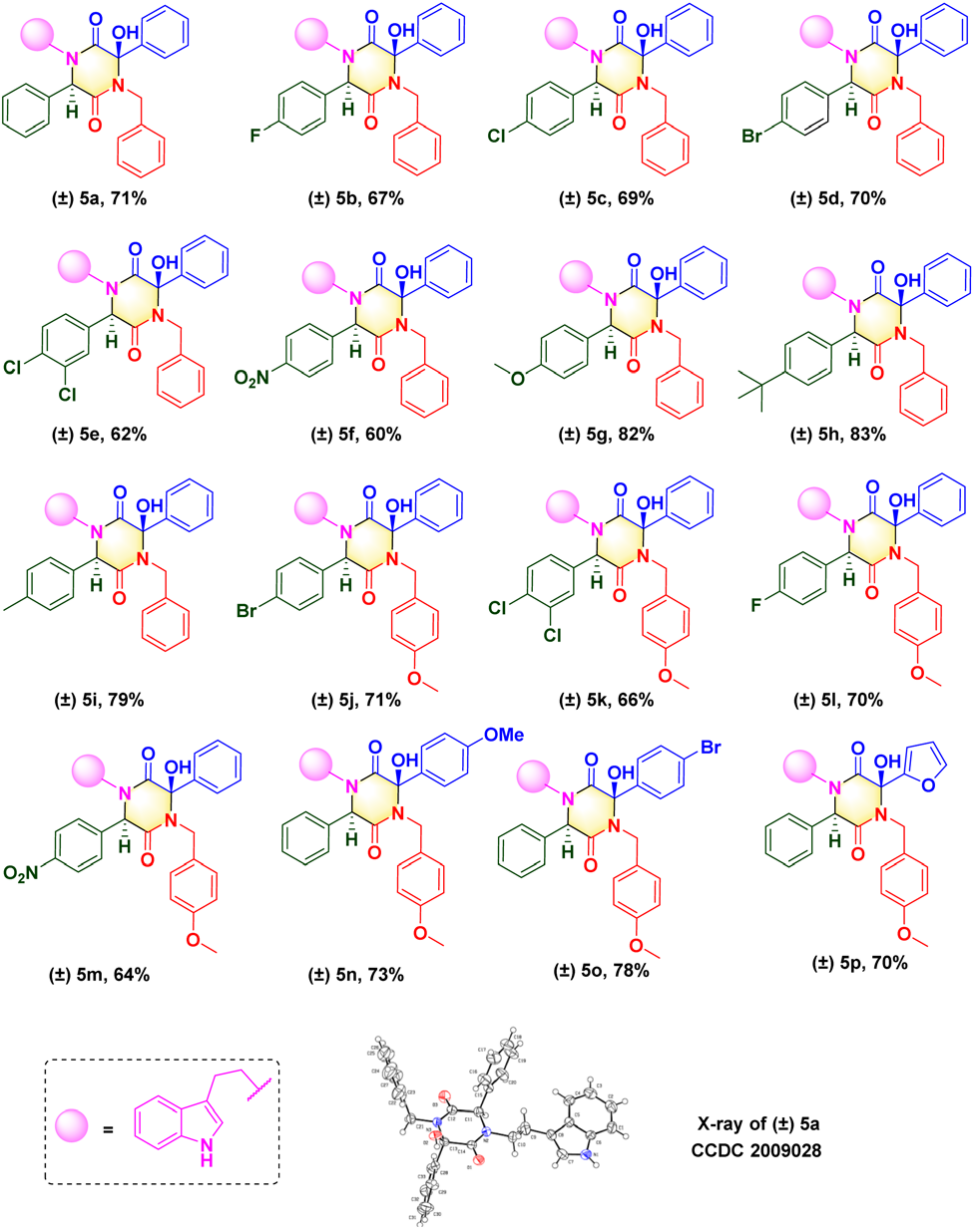
Scope of 2,5-diketopiperazines.

To gain further insights into the reaction mechanism, several control experiments were conducted (Scheme 4). The highly functionalized Ugi adduct 6 was synthesized using phenethylamine as the amine source. Regrettably, the corresponding 2,5-diketopiperazine analogue (±) 7 was not obtained under identical conditions. Subsequently, we replaced tryptamine with benzylamine or aniline to facilitate model substrates. As anticipated, desired products were not detected. These data suggested that the N–H of the indole moiety was essential to activate the ketone for the nucleophilic addition reaction (Scheme 4a). Based on control experiments and previous works, the mechanism of this green synthesis was proposed (Scheme 4b). Adduct D were generated *in situ* using methanol as the solvent at room temperature. Due to steric hindrance, the NH group of the indole exhibited a strong interaction with the ketone of the acid moiety to start the nucleophilic addition reaction through key intermediate E. Deprotonated amide F attacked the activated ketone to achieve the final compound (±) 5.

**Scheme 4:**
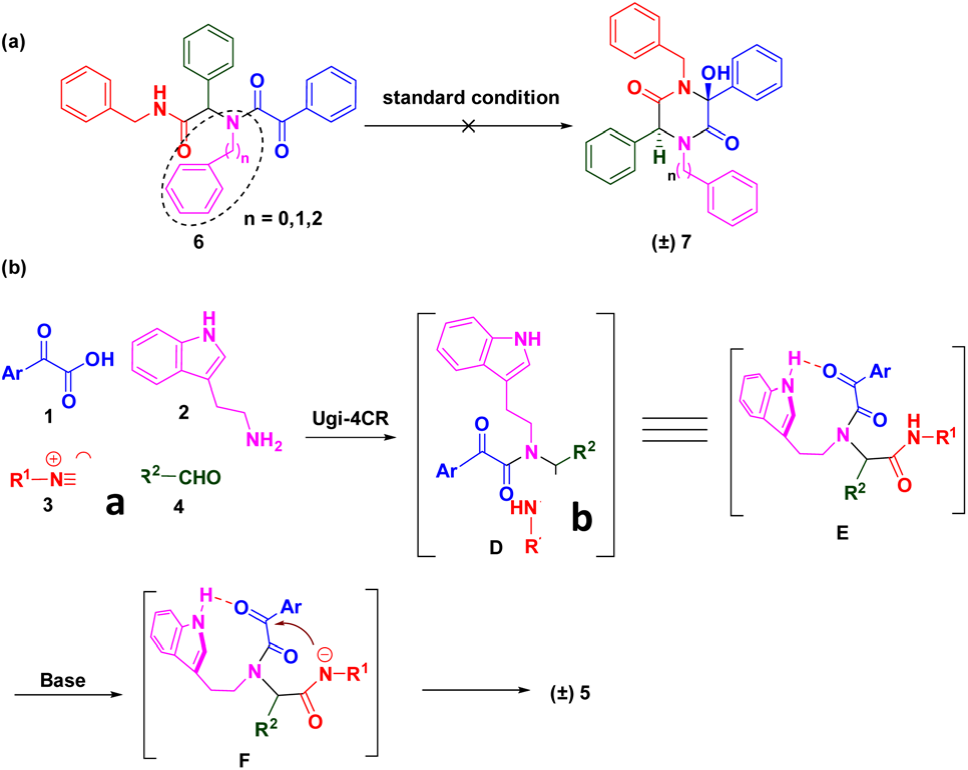
Control experiments and proposed reaction mechanism.

Biological application of 2,5-diketopiperazines was investigated for development of a lead drug compound. Synthesized compounds were evaluated for their *in vitro* toxicity against the human cancer cell lines DU145, PC3, MV411 and Jurkat by the MTT assay (Table 2). Gladly, compound (±) 5e showed higher inhibitory activity against the prostate-cancer cell lines DU 145 and PC3 at 10 μM (Fig. 2a and b). In contrast, compound (±) 5c displayed the highest toxicity against myeloid leukaemia cell lines (Fig. 2c and d). Compound (±) 5c had potent inhibitory activity against acute myeloid leukaemia (MV411, IC_50_ = 1.7 μM) and acute T lymphocyte leukaemia (Jurkat, IC_50_ = 5.7 μM) lines (ESI†). Compound (±) 5c dose-dependently induced cell-cycle arrest and apoptosis in mv411 cells (Fig. 2e and f; ESI†). Compound (±) 5c could be a promising lead compound for selective treatment of acute myeloid leukaemia. Our previous study on the synthesis of maleimide derivatives using scaffold C as a starting reagent revealed them to have broad-spectrum anticancer activity against 12 screened cancer cell lines.^17^ The newly obtained 2,5-diketopiperazines in the present study revealed selectivity against myeloid leukaemia cells (Fig. 2). To measure the cytotoxicity of these compounds, we treated a normal adult prostatic epithelial cell line (PNT1A) with these compounds at a concentration of 10 μM: cell viability was not affected.

**Table 2:** Cell viability of (±) 5

Entry	Name	DU145	MV4-11	Jurkat	PC3
1	(±) 5a	75.50	15.02	53.19	61.36
2	(±) 5b	62.58	37.09	33.85	25.48
3	(±) 5c	58.71	8.88	27.31	59.60
4	(±) 5d	54.40	10.02	30.11	54.07
5	(±) 5e	26.30	21.08	30.94	18.74
6	(±) 5f	100.78	17.28	43.14	66.43
7	(±) 5g	91.91	19.08	63.80	97.94
8	(±) 5h	74.02	15.76	37.18	78.48
9	(±) 5i	64.03	22.65	34.39	57.54
10	(±) 5j	93.17	18.92	40.33	93.37
11	(±) 5k	107.35	52.87	37.04	63.43
12	(±) 5l	68.02	17.05	47.33	67.17
13	(±) 5m	101.39	24.95	75.16	94.37
14	(±) 5o	101.35	11.96	43.29	76.40
15	(±) 5p	93.52	33.11	45.21	58.73
16	THZ1^a^	69.45	19.23	16.24	82.91

a THZ1 is a selective inhibitor of CDK7.

**Fig. 2:**
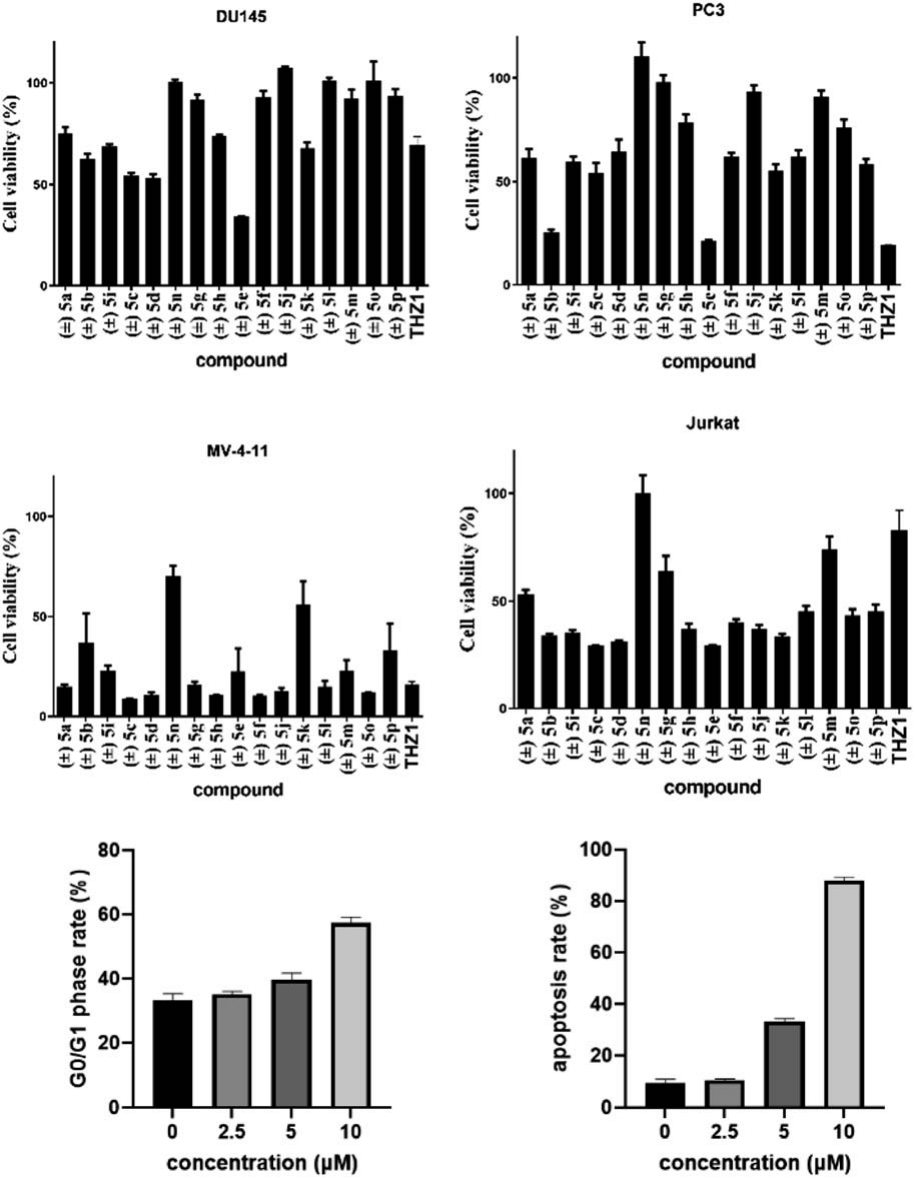
MTT assay for synthesized compounds. Cells were treated with compounds (±) 5a–p at 10 μM for 24 h, respectively, and cell viability was measured. Data are the mean ± SD of three independent experiments. Each experiment was conducted in sextuplicate.

## Conclusions

Highly functionalized Ugi adducts synthesized using tryptamine, isocyanides, glyoxylic acids and aldehydes as starting materials were subjected to a solvent-free condition. A series of 2,5-diketopiperazines was provided with good yields through an intramolecular hydrogen bond-promoted one-pot procedure. Remarkably, the products possessed potent anticancer activity *in vitro* according to the MTT assay. An in-depth study to discover the mechanism of action of compounds is underway. This green chemistry-based process provides opportunities for designing and synthesizing many compounds which may have medicinal applications.

## Ethical statement

The Laboratory Animal Welfare and Ethics Committee of Chongqing University of Arts and Sciences approved (CQWLDF0011) the study protocol on 11 March 2022. Experiments were conducted in compliance with relevant laws or guidelines set by the Chinese government.

## Conflicts of interest

There are no conflicts to declare.

## Supplementary Material

RA-012-D2RA04958A-s001

RA-012-D2RA04958A-s002
