# Correction: An intramolecular hydrogen bond-promoted “green” Ugi cascade reaction for the synthesis of 2,5-diketopiperazines with anticancer activity

**DOI:** 10.1039/d3ra90013d

**Published:** 2023-02-09

**Authors:** Jie Li, Jiu Hong Huang, Jing Ya Wang, Zhi Gang Xu, Zhong Zhu Chen, Jie Lei

**Affiliations:** a College of Pharmacy, National & Local Joint Engineering Research Center of Targeted and Innovative Therapeutics, IATTI, Chongqing University of Arts and Sciences Chongqing 402160 China

## Abstract

Correction for ‘An intramolecular hydrogen bond-promoted “green” Ugi cascade reaction for the synthesis of 2,5-diketopiperazines with anticancer activity’ by Jie Li *et al.*, *RSC Adv.*, 2022, **12**, 33175–33179, https://doi.org/10.1039/D2RA04958A.

The authors regret that it was not made clear in their original article that the X-ray crystal structure reported for product 5a, including the associated CIF file (CCDC 2009028), was not their own and was originally published by Meng Lan Lv *et al.* in 2020.^1^ The omitted reference is listed below.

The Electronic supplementary information (ESI) has been updated as there were errors in the crystallography data for 5a.

The authors sincerely apologise for these oversights.

The Royal Society of Chemistry apologises for these errors and any consequent inconvenience to authors and readers.

## Supplementary Material
