# Supplementary material for: An intramolecular hydrogen bond-promoted “green” Ugi cascade reaction for the synthesis of 2,5-diketopiperazines with anticancer activity
Source: RSC Adv. 2022 Nov 21;12(51):33175–9. doi: 10.1039/d2ra04958a (PMC9678023; doi:10.1039/d2ra04958a)
Supplement: RA-012-D2RA04958A-s001 [file RA-012-D2RA04958A-s001.pdf]

Electronic Supplementary Material (ESI) for RSC Advances.
This journal is © The Royal Society of Chemistry 2022

Supporting Information

An intramolecular hydrogen bond-promoted green Ugi cascade reaction for synthesis of 2,5-diketopiperazines with anticancer activity

Jie Li,[a] Jiu-Hong Huang,[a] Jing-Ya Wang,[a] Zhi-Gang Xu,[a] Zhong-Zhu Chen,[a] Jie Lei*[a]

[a] *College of Pharmacy, National & Local Joint Engineering Research Center of Targeted and Innovative Therapeutics, IATTI, Chongqing University of Arts and Sciences, Chongqing 402160, China.*

*Corresponding author: jlei0916@163.com

Table of Contents **Page**

General Experimental..2

General procedures for compound (±) **5**……………………………..………....2

X-ray structure and data of (±) **5a**……………………………..………...………....3

Cell lines and culture and MTT assay….…………………………………………10-12

NMR Characterization Data and Figures of Products…......................................13-39

General Experimental

^{1}H and ^{13}C NMR were recorded on a Bruker 400 spectrometer (Switzerland NMR Nuclear Magnetic Resonance Spectrometer, AVANCE II 400). ^{1}H NMR data are reported as follows: chemical shift in ppm (δ), multiplicity (s = singlet, d = doublet, t = triplet, m = multiplet), coupling constant (Hz), relative intensity. ^{13}C NMR data are reported as follows: chemical shift in ppm (δ). LC/MS analyses were performed on a Shimadzu-2020 LC-MS instrument (Japanese, Shimadzu, N2G 40-A200.6) using the following conditions: Shim-pack VP-ODS C18 column (reverse phase, 150 × 4.6 mm); a linear gradient from 10% water and 90% acetonitrile to 75% acetonitrile and 25% water over 6.0 min; flow rate of 0.5 mL/min; UV photodiode array detection from 200 to 400 nm. High-resolution mass spectra (HRMS) (Thermo Scientific Q Exactive; Shimadzu 7250, Japanese) were recorded on Thermo Scientific Exactive Plus System. The products were purified by Biotage Isolera™ Spektra Systems and hexane/EtOAc solvent systems. All reagents and solvents were obtained from commercial sources and used without further purification.

General procedures for compounds (±) 5

A mixture of acid (0.3 mmol), isocyanide (0.3 mmol), amine (0.3 mmol) and aldehyde (0.3 mmol) was stirred overnight in methanol (2.0 mL). The reaction mixture was monitored by TLC. When the reaction was completed, the solvent was removed under reduced pressure. The crude compound was directly treated with DIPA (2.0 equiv.) at MW 160 °C, 20 min. Then the reaction mixture was diluted with EtOAc (15.0 mL), washed with sat. Na_2CO_3 and brine. The organic layer was dried over $MgSO_4$ and concentrated. The residue was purified by silica gel column chromatography using a gradient of ethyl acetate/hexane (0-100%) to afford the relative targeted product.

X-ray structure and data of (±)5a

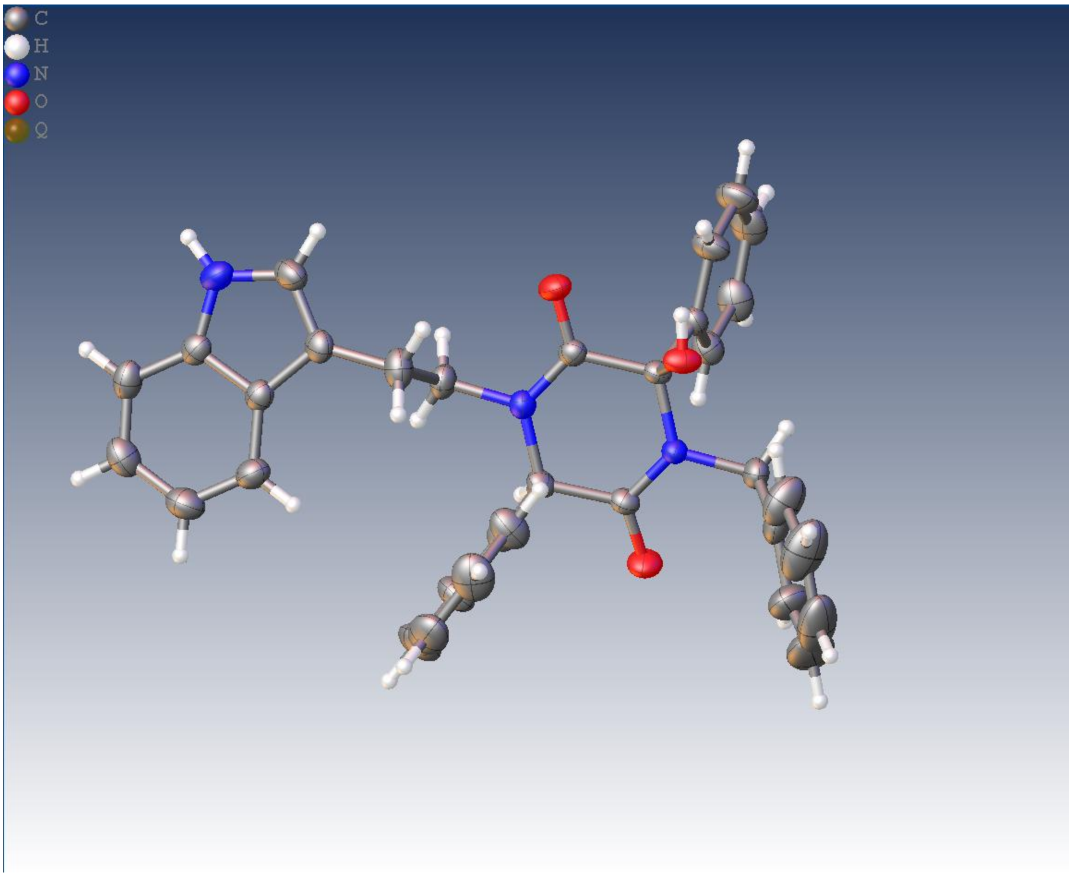

Table 1 Crystal data and structure refinement for 1.

Identification code	1
Empirical formula	CHNO
Formula weight	38.19
Temperature/K	296.15
Crystal system	triclinic
Space group	P-1
a/Å	7.1229(8)
b/Å	12.8851(13)
c/Å	15.0472(15)
α/°	92.354(2)
β/°	103.338(2)
γ/°	91.502(2)

Volume/Å^3	1341.7(2)
Z	27
ρ_{calc}g/cm^3	1.2762
μ/mm^{-1}	0.083
F(000)	544.2
Radiation	Mo Kα (λ = 0.71073)
2Θ range for data collection/°	3.16 to 55.02
Index ranges	-9 ≤ h ≤ 7, -14 ≤ k ≤ 16, -19 ≤ l ≤ 19
Reflections collected	8108
Independent reflections	5823 [R_{int} = 0.0182, R_{sigma} = 0.0286]
Data/restraints/parameters	5823/0/354
Goodness-of-fit on F^2	1.058
Final R indexes [I>=2σ (I)]	R_1 = 0.0422, wR_2 = 0.1188
Final R indexes [all data]	R_1 = 0.0571, wR_2 = 0.1367
Largest diff. peak/hole / e Å^{-3}	0.31/-0.23

Table 2 Fractional Atomic Coordinates (×10^4) and Equivalent Isotropic Displacement Parameters (Å^2×10^3) for 1. U_{eq} is defined as 1/3 of of the trace of the orthogonalised U_{IJ} tensor.

Atom	*x*	*y*	*z*	U(eq)
O001	11250.2(13)	2290.4(8)	3661.1(6)	34.9(2)
O002	5012.6(14)	2169.7(8)	3627.1(7)	42.0(3)
O003	10558.6(16)	3168.5(8)	1928.5(7)	41.0(3)
N004	8040.8(15)	1937.7(8)	3472.9(7)	26.4(2)
N005	7664.0(17)	3530.4(8)	2211.7(7)	30.0(3)
N006	8154(2)	6912.3(10)	-254.1(9)	42.0(3)
C007	9563.1(17)	2074.6(10)	2978.2(8)	24.9(3)
C008	9630.7(19)	1101.8(10)	2357.5(9)	28.3(3)
C009	6377.0(18)	2435.5(10)	3299.7(9)	27.6(3)
C00A	9263.5(19)	2987.7(10)	2330.3(9)	27.9(3)
C00B	6524(2)	7221.2(10)	9.9(9)	32.1(3)

C00C	6146.0(18)	3375.4(10)	2710.5(9)	28.6(3)
C00D	5943(2)	4342.6(11)	3294.8(9)	33.0(3)
C00E	8224(2)	1053.4(10)	4094.6(9)	33.7(3)
C00F	6442(2)	6705.0(10)	813.1(9)	31.0(3)
C00G	7968(2)	494.4(11)	2001.6(10)	36.8(3)
C00H	8100(2)	6068.8(10)	1020.3(10)	35.3(3)
C00I	5113(2)	7908.1(11)	-379.1(10)	40.2(4)
C00J	7340(2)	4321.3(11)	1511.6(10)	35.7(3)
C00K	4912(2)	6894.8(12)	1230.8(10)	40.2(4)
C00L	11306(2)	870.6(12)	2080.2(11)	42.6(4)
C00M	9076(2)	6220.9(11)	352.6(11)	42.0(4)
C00N	3617(2)	8067.2(12)	40.9(11)	46.0(4)
C00O	8565(3)	1388.4(11)	5095.8(10)	42.5(4)
C00P	8563(2)	5330.0(11)	1779.2(11)	42.9(4)
C00Q	4178(2)	4798.7(13)	3190.8(13)	48.5(4)
C00R	9640(3)	-532.0(13)	1084.5(11)	51.8(4)
C00S	7514(3)	4777.9(14)	3930.1(11)	50.7(4)
C00T	7977(3)	-326.0(12)	1372.3(11)	47.5(4)
C00U	3523(2)	7573.4(13)	841.4(12)	47.8(4)
C00V	11295(3)	58.6(14)	1436.9(13)	54.2(5)
C00W	10354(4)	1761.5(17)	5579.1(13)	67.6(6)
C00X	7310(3)	5660.0(15)	4458.4(13)	63.5(5)
C00Y	3997(3)	5684.6(15)	3723.3(16)	65.0(5)
C00Z	5549(4)	6105.9(14)	4350.4(14)	64.4(6)
C010	7105(4)	1296.1(18)	5557.7(14)	71.5(6)
C011	7469(5)	1585(2)	6494.2(18)	96.9(10)
C012	9259(6)	1962.7(19)	6952.2(16)	94.2(9)
C013	10678(5)	2049(2)	6508.0(15)	91.7(9)

Table 3 Anisotropic Displacement Parameters (Å^2×10^3) for 1. The Anisotropic displacement factor exponent takes the form: $-2\pi^2[h^2a^{*2}U_{11}+2hka^*b^*U_{12}+...]$.

Atom	U_{11}	U_{22}	U_{33}	U_{12}	U_{13}	U_{23}
O001	22.2(5)	51.6(6)	31.0(5)	0.3(4)	7.7(4)	-4.4(4)
O002	25.6(5)	50.5(6)	54.6(6)	4.2(5)	17.2(5)	12.0(5)
O003	45.8(6)	37.5(5)	49.1(6)	4.1(5)	29.2(5)	7.3(4)
N004	24.4(5)	27.0(5)	30.2(5)	4.6(4)	10.3(4)	5.7(4)
N005	33.5(6)	27.8(6)	30.4(6)	5.2(5)	9.8(5)	4.9(4)
N006	55.1(8)	36.3(6)	42.2(7)	2.5(6)	26.6(6)	4.1(5)
C007	21.0(6)	27.9(6)	26.9(6)	3.2(5)	7.6(5)	0.1(5)
C008	32.1(7)	25.6(6)	27.4(6)	7.0(5)	6.8(5)	2.2(5)
C009	22.2(6)	29.7(6)	31.0(6)	1.8(5)	6.7(5)	-0.7(5)
C00A	31.7(7)	25.3(6)	28.4(6)	0.8(5)	10.9(5)	-1.3(5)
C00B	40.7(8)	26.4(6)	29.9(7)	-2.6(6)	10.2(6)	-1.9(5)
C00C	23.4(6)	31.3(7)	30.7(7)	5.6(5)	4.5(5)	1.8(5)
C00D	35.3(7)	30.9(7)	34.6(7)	7.2(6)	11.0(6)	2.8(5)
C00E	37.4(8)	27.6(7)	38.8(7)	4.1(6)	12.9(6)	8.3(6)
C00F	37.5(7)	25.5(6)	30.2(7)	-1.1(5)	8.7(6)	0.2(5)
C00G	36.0(8)	35.3(7)	36.6(7)	4.7(6)	3.2(6)	-0.9(6)
C00H	39.9(8)	24.7(6)	41.3(8)	0.3(6)	9.7(6)	2.0(6)
C00I	54.2(9)	32.1(7)	30.8(7)	0.8(7)	2.4(7)	3.5(6)
C00J	44.0(8)	30.7(7)	32.0(7)	4.0(6)	6.3(6)	7.7(5)
C00K	45.3(9)	40.6(8)	38.9(8)	0.5(7)	18.0(7)	3.2(6)
C00L	39.8(8)	39.4(8)	51.9(9)	4.4(7)	18.6(7)	-8.4(7)
C00M	43.6(9)	30.2(7)	57.0(9)	5.8(6)	21.5(7)	0.6(6)
C00N	42.8(9)	41.3(8)	49.5(9)	9.0(7)	1.1(7)	-0.4(7)
C00O	60.7(10)	33.1(7)	37.9(8)	11.0(7)	17.2(7)	12.9(6)
C00P	48.5(9)	30.9(7)	45.2(8)	2.2(7)	1.7(7)	6.1(6)
C00Q	38.7(9)	41.2(8)	67.4(11)	10.7(7)	15.9(8)	-1.3(8)
C00R	75.8(13)	36.1(8)	42.5(9)	10.0(8)	12.8(8)	-10.7(7)
C00S	48.9(10)	51.0(10)	46.1(9)	10.0(8)	-0.4(8)	-8.6(7)
C00T	55.9(10)	38.0(8)	41.4(8)	-0.2(7)	-1.8(7)	-7.6(7)
C00U	40.7(9)	49.3(9)	56.6(10)	6.4(7)	18.5(8)	-2.6(8)
C00V	61.5(11)	48.3(9)	59.6(10)	11.3(8)	29.4(9)	-11.3(8)
C00W	86.4(15)	70.8(13)	40.7(9)	-15.1(11)	5.9(10)	9.3(9)
C00X	83.3(15)	53.1(11)	46.9(10)	1.5(10)	3.4(10)	-13.3(8)
C00Y	63.7(12)	48.0(10)	91.9(15)	21.5(9)	34.6(12)	-3.9(10)
C00Z	98.5(16)	39.6(9)	63.2(12)	9.9(10)	36.8(12)	-8.2(8)
C010	84.1(16)	86.8(15)	57.0(12)	21.3(12)	38.5(11)	22.1(11)
C011	143(3)	109(2)	62.8(15)	43(2)	64.0(18)	25.0(14)
C012	175(3)	68.3(15)	41.6(11)	24.8(18)	26.8(17)	11.6(10)
C013	140(3)	81.1(16)	44.4(11)	-18.0(16)	3.1(14)	6.0(11)

Table 4 Bond Lengths for 1.

Atom	Atom	Length/Å	Atom	Atom	Length/Å
O001	C007	1.4024(15)	C00F	C00H	1.438(2)
O002	C009	1.2337(16)	C00F	C00K	1.400(2)
O003	C00A	1.2363(16)	C00G	C00T	1.391(2)
N004	C007	1.4609(15)	C00H	C00M	1.364(2)
N004	C009	1.3397(16)	C00H	C00P	1.500(2)
N004	C00E	1.4931(16)	C00I	C00N	1.374(2)
N005	C00A	1.3331(17)	C00J	C00P	1.532(2)
N005	C00C	1.4655(16)	C00K	C00U	1.381(2)
N005	C00J	1.4799(16)	C00L	C00V	1.394(2)
N006	C00B	1.3737(19)	C00N	C00U	1.400(2)
N006	C00M	1.371(2)	C00O	C00W	1.377(3)
C007	C008	1.5405(17)	C00O	C010	1.383(3)
C007	C00A	1.5465(17)	C00Q	C00Y	1.394(2)
C008	C00G	1.388(2)	C00R	C00T	1.380(3)
C008	C00L	1.388(2)	C00R	C00V	1.372(3)
C009	C00C	1.5202(18)	C00S	C00X	1.390(2)
C00B	C00F	1.4153(19)	C00W	C013	1.396(3)
C00B	C00I	1.398(2)	C00X	C00Z	1.372(3)
C00C	C00D	1.5249(18)	C00Y	C00Z	1.361(3)
C00D	C00Q	1.381(2)	C010	C011	1.405(3)
C00D	C00S	1.382(2)	C011	C012	1.368(4)
C00E	C00O	1.512(2)	C012	C013	1.339(4)

Table 5 Bond Angles for 1.

Atom	Atom	Atom	Angle/°	Atom	Atom	Atom	Angle/°
C009	N004	C007	124.46(10)	C00H	C00F	C00B	107.03(12)
C00E	N004	C007	116.63(10)	C00K	C00F	C00B	118.98(13)
C00E	N004	C009	118.03(10)	C00K	C00F	C00H	133.99(13)
C00C	N005	C00A	124.34(11)	C00T	C00G	C008	120.59(15)
C00J	N005	C00A	118.77(11)	C00M	C00H	C00F	106.07(13)
C00J	N005	C00C	116.86(11)	C00P	C00H	C00F	126.33(13)
C00M	N006	C00B	108.71(12)	C00P	C00H	C00M	127.44(14)
N004	C007	O001	104.82(10)	C00N	C00I	C00B	117.84(14)
C008	C007	O001	114.62(10)	C00P	C00J	N005	114.96(12)
C008	C007	N004	110.07(10)	C00U	C00K	C00F	118.91(14)
C00A	C007	O001	108.39(10)	C00V	C00L	C008	120.23(15)
C00A	C007	N004	114.02(10)	C00H	C00M	N006	110.70(14)
C00A	C007	C008	105.16(10)	C00U	C00N	C00I	121.25(15)
C00G	C008	C007	120.32(12)	C00W	C00O	C00E	120.90(16)
C00L	C008	C007	120.63(12)	C010	C00O	C00E	120.87(17)
C00L	C008	C00G	118.81(12)	C010	C00O	C00W	118.20(18)
N004	C009	O002	121.72(12)	C00J	C00P	C00H	109.99(12)
C00C	C009	O002	117.96(11)	C00Y	C00Q	C00D	120.04(17)
C00C	C009	N004	120.29(11)	C00V	C00R	C00T	119.81(14)
N005	C00A	O003	122.77(12)	C00X	C00S	C00D	120.27(17)
C007	C00A	O003	116.59(11)	C00R	C00T	C00G	120.06(16)
C007	C00A	N005	120.63(11)	C00N	C00U	C00K	121.27(15)
C00F	C00B	N006	107.49(13)	C00R	C00V	C00L	120.46(16)
C00I	C00B	N006	130.78(13)	C013	C00W	C00O	120.9(2)
C00I	C00B	C00F	121.74(13)	C00Z	C00X	C00S	120.17(19)
C009	C00C	N005	114.76(10)	C00Z	C00Y	C00Q	120.52(18)
C00D	C00C	N005	111.38(11)	C00Y	C00Z	C00X	119.93(16)
C00D	C00C	C009	109.38(10)	C011	C010	C00O	119.9(3)
C00Q	C00D	C00C	120.12(13)	C012	C011	C010	120.4(2)
C00S	C00D	C00C	120.81(13)	C013	C012	C011	119.8(2)
C00S	C00D	C00Q	119.07(14)	C012	C013	C00W	120.8(3)
C00O	C00E	N004	113.80(11)				

Table 6 Hydrogen Atom Coordinates (Å×10^4) and Isotropic Displacement Parameters (Å^2×10^3) for 1.

Atom	*x*	*y*	*z*	U(eq)
H00L	12441(2)	1258.6(12)	2324.3(11)	51.2(4)
H00V	12417(3)	-83.5(14)	1245.6(13)	65.0(5)
H00R	9638(3)	-1069.7(13)	652.7(11)	62.2(5)
H00T	6862(3)	-736.1(12)	1145.4(11)	57.0(5)
H00G	6838(2)	637.1(11)	2185.4(10)	44.2(4)
H010	5884(4)	1043.1(18)	5249.1(14)	85.8(7)
H011	6488(5)	1519(2)	6804.4(18)	116.3(11)
H012	9489(6)	2159.2(19)	7570.6(16)	113.0(11)
H013	11895(5)	2303(2)	6822.3(15)	110.1(10)
H00W	11359(4)	1823.0(17)	5282.8(13)	81.1(7)
H00S	8711(3)	4479.5(14)	4004.6(11)	60.8(5)
H00X	8371(3)	5948.6(15)	4886.2(13)	76.2(6)
H00Z	5416(4)	6695.4(14)	4705.2(14)	77.2(7)
H00Y	2806(3)	5990.4(15)	3649.7(16)	78.0(7)
H00Q	3109(2)	4514.5(13)	2765.2(13)	58.2(5)
H00K	4832(2)	6569.2(12)	1761.9(10)	48.3(4)
H00U	2504(2)	7704.8(13)	1116.4(12)	57.3(5)
H00N	2650(2)	8511.4(12)	-211.6(11)	55.2(5)
H00I	5184(2)	8246.4(11)	-905.2(10)	48.3(4)
H001	12191(2)	2269(14)	3434(2)	52.3(4)
H006	8533(2)	7118.5(10)	-722.6(9)	50.3(4)
H00C	4925.6(18)	3265.2(10)	2252.1(9)	34.4(3)
H00a	7053(2)	617.8(10)	3925.2(9)	40.5(4)
H00b	9286(2)	636.2(10)	4006.6(9)	40.5(4)
H00d	7608(2)	4020.0(11)	954.4(10)	42.9(4)
H00e	5988(2)	4490.3(11)	1377.8(10)	42.9(4)
H00M	10210(2)	5899.5(11)	314.3(11)	50.4(4)
H00f	9922(2)	5177.4(11)	1901.7(11)	51.5(4)
H00h	8301(2)	5647.3(11)	2331.7(11)	51.5(4)

Cell lines and culture

The human prostate tumor cells PC3 and DU145, acute lymphoblastic leukemia cells Jurkat, biphenotypic B-myelomonocytic leukemia cells MV-4-11 were obtained from American Type Culture Collection (ATCC, Manassas, VA, USA). The DU145 cells were cultured in high-glucose DMEM (Hyclone, SH30022.01, USA) medium supplemented with 10% fetal bovine serum (FBS, Gibco, 10099, Australia origin). The PC3 cells were cultured with the Ham's F-12K (Kaighn's) Medium (GIBCO, 21127022, USA) supplemented with 10% FBS. The Jurkat and MV-4-11 cells were cultured with RPMI 1640 medium (GIBCO, 11875093, USA) supplemented with 10% fetal bovine serum (FBS,Gibco, 10099,Australia). The cells were cultured in the incubator at the 37 °C and 5% CO_2 with humidified atmosphere.

Cell viability assay

The tumor cells were counted and seeded into the 96-well plate containing 100 µL complete medium, the density of cells were 3×10^3 cells per well for PC3 andDU145. After incubation for 24 hours, added another 100 µL complete medium containing 10 µM compounds **(±) 5** or equal amount of Dimethyl sulfoxide (DMSO), each treatment was triple replicated. The compound-treated cells were cultured for another 48 hours, 3-(4, 5-dimethyl-2-thiazolyl)-2, 5-diphenyl-2-*H*-tetrazolium bromide (MTT, Beyotime, ST316, Shanghai, China) was added, the plate was incubated for another 4 hours. After incubation, removed the medium and added 150 µL DMSO into each well to dissolve the formazan. The optical density (OD) of each well was measured with a microplate reader (Bio-Tek, Winooski, VT, USA) at an absorbance wavelength of 570 nm. Similar to PC3 cells, Jurkat cells and MV-4-11 cells were seeded into the 96-well plate with a density of 6×10^3 cells per well, compound-treated cells were cultured for 72 hours and followed by Cell Counting Kit-8 (CCK-8, Beyotime, C0037, Shanghai, China) treatment for another 4 hours. The OD was measured at an absorbance wavelength of 450 nm. The viability of compounds 5 treated cells equal to the ration of $OD_{compound}$ to OD_{DMSO}. To further evaluate the half maximal inhibitory concentration (IC_{50}) of compound **(±) 5c**, MV-4-11 and Jurkat cells were incubated with various concentrations (0, 0.625, 1.25, 2.5, 5, 10 µM) of compound **(±) 5c** for 72 h. The OD of compound-treated cells was measured with CCK-8. The IC_{50} values were analyzed by GraphPad Prism 8.

Transform of (±) 5c inhibits MV411 IC_{50}

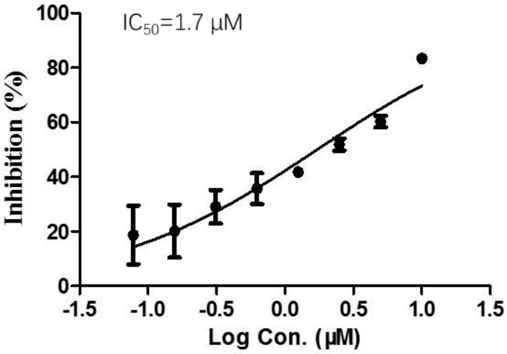

Transform of (±) 5e IC_{50} in Jurkat

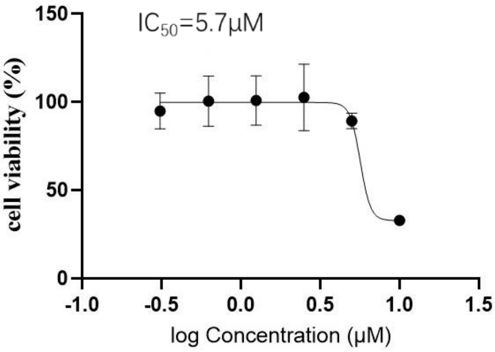

Flow cytometry analysis

Leukemia cells MV4-11 in the logarithmic growth condition were harvested and seeded into 6-well plates at concentration of 500 thousand per well. The cells were treated with different concentrations of compound (±) **5 e** or the same amount of DMSO for 48 hours. All cells were collected and analyzed using flow cytometry analysis. For cell cycle analysis, cell were fixed with 70% ethanol at 4 °C for 24 hours, and then washed with PBS for 3 times. Subsequently, cells were stained with PI (50mg/ml, BD Biosciences) and RNase (100mg/ml, Sigma-Aldrich) at 37℃ for 30 minutes. The stained cells were analyzed with flow cytometry (Accuri C6, BD biosciences) and the results were recorded. For apoptosis analysis, cells were collected and stained with an Annexin V-FITC/PI apoptosis assay kit following the manufacture’s manual (BD Biosciences). The stained cells were detected with flow cytometry (Accuri C6, BD biosciences). All flow cytometry analysis results were visualized using FlowJo 7.6. The statistical chart of each treatment represents mean±SD of 3 (n=3).

(±) 5c induces apoptosis in MV411			
**0 μM**	**2.5 μM**	**5 μM**	**10 μM**
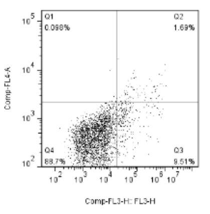	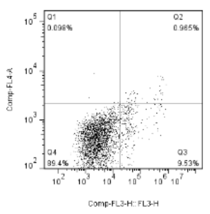	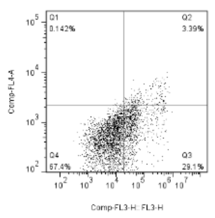	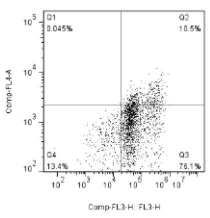

(±) 5c induces cell cycle arrest in MV411			
**0 µM**	**2.5 µM**	**5 µM**	**10 µM**
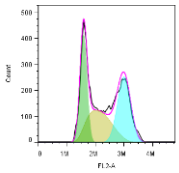	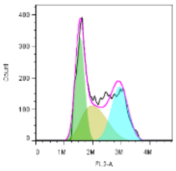	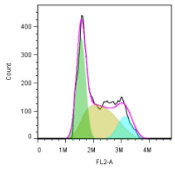	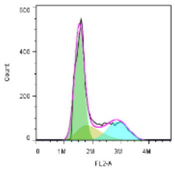

NMR Characterization Data and Figures of Products

1-(2-(1H-indol-3-yl)ethyl)-4-benzyl-3-hydroxy-3,6-diphenylpiperazine-2,5-dione

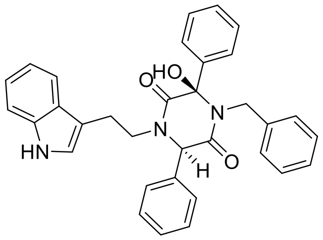

(±) 5a, brown oil, yield 71%, (EA/Hex = 30%, R_f = 0.3), purity 97%. ^{1}H NMR (400 MHz, $CDCl_3$) δ 7.65 (s, 1H), 7.53 (d, *J* = 7.2 Hz, 2H), 7.47 (d, *J* = 7.9 Hz, 1H), 7.39 – 7.28 (m, 4H), 7.24 – 7.20 (m, 1H), 7.18 – 7.09 (m, 6H), 7.06-7.00 (m, 3H), 6.82 (d, *J* = 7.7 Hz, 2H), 6.77 (s, 1H), 5.48 (s, 1H), 4.93 (d, *J* = 14.1 Hz, 1H), 4.85 (s, 1H), 4.25 (d, *J* = 14.1 Hz, 1H), 4.22-4.15 (m, 1H), 3.22 – 3.14 (m, 1H), 3.06 – 3.00 (m, 1H), 2.96 – 2.89 (m, 1H). ^{13}C NMR (101 MHz, $CDCl_3$) δ 167.95, 165.27, 139.35, 137.93, 136.24, 133.01, 129.36, 129.00, 128.39, 128.17, 127.27, 126.69, 126.28, 122.37, 119.63, 118.32, 111.39, 85.33, 64.85, 47.59, 22.64. HRMS (ESI) m/z calcd for $C_{33}H_{30}N_3O_3^+$ $(M+H)^+$ 516.2287, found 516.2279.

1-(2-(1H-indol-3-yl)ethyl)-4-benzyl-6-(4-fluorophenyl)-3-hydroxy-3-phenylpiperazine-2,5-dione

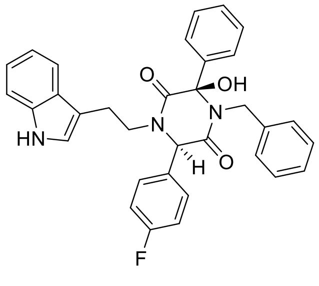

(±) 5b, brown oil, yield 67% (EA/Hex = 30%, R_f = 0.3), purity 95%. ^{1}H NMR (400 MHz, $CDCl_3$) δ 7.62 (s, 1H), 7.53 (d, *J* = 7.2 Hz, 2H), 7.47 (d, *J* = 7.9 Hz, 1H), 7.42 – 7.32 (m, 3H), 7.29 (d, *J* = 8.3 Hz, 1H), 7.25 (s, 1H), 7.18-7.13 (m, 3H), 7.08 (d, *J* = 7.6 Hz, 2H), 7.02 (t, *J* = 7.5 Hz, 1H), 6.82 – 6.66 (m, 5H), 5.50 (s, 1H), 4.93 (d, *J* = 14.1 Hz, 1H), 4.77 (s, 1H), 4.28 (d, *J* = 14.1 Hz, 1H), 4.21-4.14 (m, 1H), 3.22 – 3.14

(m, 1H), 3.06 – 3.00 (m, 1H), 2.95 – 2.87 (m, 1H). ^{13}C NMR (101 MHz, $CDCl_3$) δ 167.94, 165.17, 139.38, 137.83, 136.25, 129.39, 128.49, 128.20, 127.34, 126.80, 126.18, 122.41, 119.68, 118.25, 115.24, 111.45, 85.22, 64.31, 47.56, 47.35, 22.66. HRMS (ESI) m/z calcd for $C_{33}H_{29}FN_3O_3^+$ $(M+H)^+$ 534.2193, found 534.2199.

1-(2-(1H-indol-3-yl)ethyl)-4-benzyl-6-(4-chlorophenyl)-3-hydroxy-3-phenylpiperazine-2,5-dione

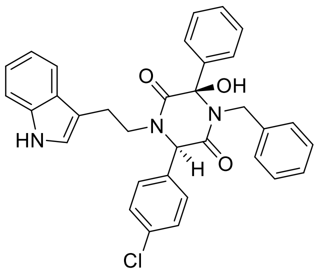

(±) 5c, brown oil, yield 69%, (EA/Hex = 30%, R_f = 0.3), purity 97%. ^{1}H NMR (400 MHz, $CDCl_3$) δ 7.62 (s, 1H), 7.52 (d, *J* = 7.3 Hz, 2H), 7.46 (d, *J* = 7.9 Hz, 1H), 7.41 – 7.32 (m, 3H), 7.29 (d, *J* = 8.4 Hz, 1H), 7.24 (s, 1H), 7.18-7.13 (m, 3H), 7.07 (d, *J* = 7.6 Hz, 2H), 7.04-6.98 (m, 3H), 6.75-6.69 (m, 3H), 5.50 (s, 1H), 4.92 (d, *J* = 14.1 Hz, 1H), 4.74 (s, 1H), 4.28 (d, *J* = 14.1 Hz, 1H), 4.21 – 4.12 (m, 1H), 3.23 – 3.13 (m, 1H), 3.07 – 2.98 (m, 1H), 2.95 – 2.85 (m, 1H). ^{13}C NMR (101 MHz, $CDCl_3$) δ 167.94, 164.92, 139.33, 137.79, 136.25,134.19, 131.55, 129.39, 128.52, 127.98, 127.36, 126.77, 126.15, 122.43, 119.70, 118.23, 111.46, 85.22, 64.40, 47.56, 47.37, 22.66. HRMS (ESI) m/z calcd for $C_{33}H_{29}ClN_3O_3^+$ $(M+H)^+$ 550.1897, found 550.1889.

1-(2-(1H-indol-3-yl)ethyl)-4-benzyl-6-(4-bromophenyl)-3-hydroxy-3-phenylpiperazine-2,5-dione

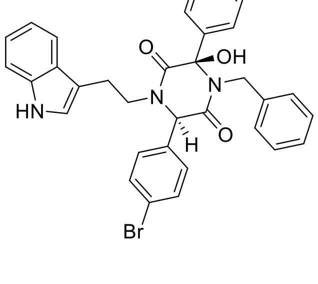

(±) 5d, brown oil, yield 70%, (EA/Hex = 30%, R_f = 0.3), purity 97%. ^{1}H NMR (400 MHz, $CDCl_3$) δ 7.62 (s, 1H), 7.52 (d, *J* = 7.1 Hz, 2H), 7.46 (d, *J* = 7.9 Hz, 1H), 7.41 – 7.32 (m, 3H), 7.29 (d, *J* = 8.3 Hz, 1H), 7.25 (d, *J* = 3.5 Hz, 1H), 7.21 – 7.11 (m, 5H), 7.10 – 6.98 (m, 3H), 6.74 (d, *J* = 1.8 Hz, 1H), 6.64 (d, *J* = 8.4 Hz, 2H), 5.50 (s, 1H), 4.92 (d, *J* = 14.0 Hz, 1H), 4.71 (s, 1H), 4.28 (d, *J* = 14.1 Hz, 1H), 4.23 – 4.12 (m, 1H), 3.24 – 3.12 (m, 1H), 3.08 – 2.97 (m, 1H), 2.95 – 2.85 (m, 1H). ^{13}C NMR (101 MHz, $CDCl_3$) δ 167.94, 164.82, 139.31, 137.78, 136.25, 132.11, 131.28, 129.40, 128.53, 128.29, 127.36, 126.77, 126.14, 122.43, 119.70, 118.23, 111.46, 85.22, 64.47, 47.56, 47.37, 22.66. HRMS (ESI) m/z calcd for $C_{33}H_{29}BrN_3O_3^+$ $(M+H)^+$ 594.1392, found 594.1395.

1-(2-(1H-indol-3-yl)ethyl)-4-benzyl-6-(3,4-dichlorophenyl)-3-hydroxy-3-phenylpiperazine-2,5-dione

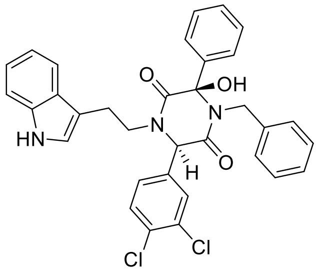

(±) 5e, red-brown oil, yield 62%, (EA/Hex = 30%, R_f = 0.3), purity 94%. ^{1}H NMR (400 MHz, $CDCl_3$) δ 7.60 (s, 1H), 7.53 (d, *J* = 7.3 Hz, 2H), 7.46 (d, *J* = 7.9 Hz, 1H), 7.41 – 7.33 (m, 3H), 7.29 (s, 1H), 7.25 (s, 1H), 7.16 (t, *J* = 7.7 Hz, 3H), 7.08 (t, *J* = 8.4 Hz, 3H), 7.02 (t, *J* = 7.5 Hz, 1H), 6.71 (s, 2H), 6.67-6.65 (m, 1H), 5.56 (s, 1H), 4.93 (d, *J* = 14.0 Hz, 1H), 4.65 (s, 1H), 4.33 (d, *J* = 14.0 Hz, 1H), 4.24 – 4.15 (m, 1H), 3.25 – 3.13 (m, 1H), 3.08 – 2.97 (m, 1H), 2.94 – 2.83 (m, 1H). ^{13}C NMR (101 MHz, $CDCl_3$) δ 168.01, 164.53, 139.18, 137.69, 136.27, 133.24, 132.57, 130.05, 129.46, 128.62, 128.25, 127.44, 126.68, 126.04, 122.49, 119.74, 118.17, 111.52, 111.14, 85.16, 64.07, 47.56, 47.44. HRMS (ESI) m/z calcd for $C_{33}H_{28}Cl_2N_3O_3^+$ $(M+H)^+$ 584.1508, found 584.1409.

1-(2-(1H-indol-3-yl)ethyl)-4-benzyl-3-hydroxy-6-(4-nitrophenyl)-3-phenylpiperazine-2,5-dione

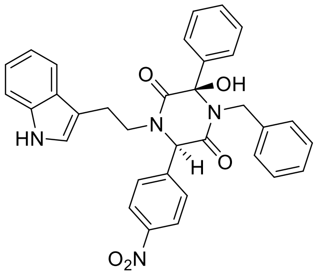

(±) 5f, red oil, yield 60%, (EA/Hex = 30%, R_f = 0.3), purity 95%. ^{1}H NMR (400 MHz, $CDCl_3$) δ 7.81 (d, *J* = 8.7 Hz, 2H), 7.64 (s, 1H), 7.54 (d, *J* = 7.1 Hz, 2H), 7.46 – 7.35 (m, 4H), 7.30 (d, *J* = 8.2 Hz, 1H), 7.23 (t, *J* = 7.3 Hz, 1H), 7.17 (t, *J* = 7.6 Hz, 1H), 7.09 (t, *J* = 7.7 Hz, 2H), 7.05 – 6.98 (m, 3H), 6.90 (d, *J* = 8.7 Hz, 2H), 6.71 (d, *J* = 2.0 Hz, 1H), 5.61 (s, 1H), 4.93 (d, *J* = 14.0 Hz, 1H), 4.76 (s, 1H), 4.35 (d, *J* = 14.0 Hz, 1H), 4.30 – 4.20 (m, 1H), 3.26 – 3.16 (m, 1H), 3.10 – 3.01 (m, 1H), 2.94 – 2.88 (m, 1H). ^{13}C NMR (101 MHz, $CDCl_3$) δ 168.04, 164.19, 147.54, 140.19, 139.27, 137.58, 136.30, 129.46, 128.60, 128.28, 127.51, 126.62, 125.94, 123.11, 122.59, 119.75, 118.09, 111.58, 110.99, 85.11, 64.75, 47.57, 22.72.HRMS (ESI) m/z calcd for $C_{33}H_{29}N_4O_5^+$ $(M+H)^+$ 561.2138, found 561.2095.

1-(2-(1H-indol-3-yl)ethyl)-4-benzyl-3-hydroxy-6-(4-methoxyphenyl)-3-phenylpiperazine-2,5-dione

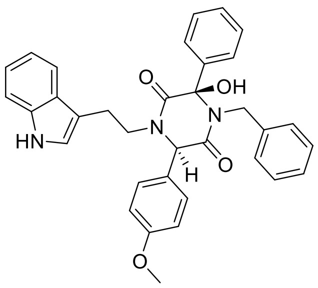

(±) 5g, brown-yellow oil, yield 82%, (EA/Hex =3 0%, R_f = 0.3), purity 97%. ^{1}H NMR (400 MHz, $CDCl_3$) δ 7.67 (s, 1H), 7.52 (d, *J* = 7.2 Hz, 2H), 7.47 (d, *J* = 7.9 Hz, 1H), 7.40 – 7.28 (m, 4H), 7.25 (m, 1H), 7.19-7.13 (m, 5H), 7.02 (t, *J* = 7.4 Hz, 1H), 6.78 (d, *J* = 1.9 Hz, 1H), 6.73 (d, *J* = 8.6 Hz, 2H), 6.58 (d, *J* = 8.7 Hz, 2H), 5.45 (s, 1H), 4.92 (d, *J* = 14.1 Hz, 1H), 4.80 (s, 1H), 4.23 (d, *J* = 14.1 Hz, 1H), 4.18 – 4.09 (m, 1H), 3.71 (s, 3H), 3.22 – 3.11 (m, 1H), 3.08 – 2.91 (m, 2H). ^{13}C NMR (101 MHz, $CDCl_3$) δ

167.85, 165.53, 159.53, 139.51, 137.95, 136.24, 129.33, 129.01, 128.44, 128.03, 127.25, 126.91, 126.35, 124.99, 122.34, 119.63, 118.35, 113.71, 111.59, 85.35, 64.38, 55.31, 47.60, 47.26, 22.64. HRMS (ESI) m/z calcd for $C_{34}H_{32}N_3O_4^+$ $(M+H)^+$ 546.2393, found 546.2356.

1-(2-(1H-indol-3-yl)ethyl)-4-benzyl-6-(4-(tert-butyl)phenyl)-3-hydroxy-3-phenylpiperazine-2,5-dione

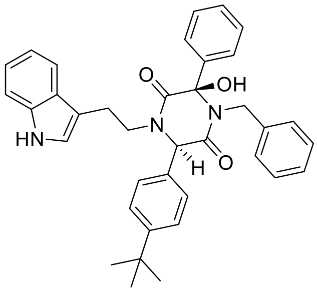

(±) 5h, brown oil, yield 83%, (EA/Hex = 30%, R_f = 0.3), purity 96%. ^1H NMR (400 MHz, $CDCl_3$) δ 7.71 (s, 1H), 7.53 (d, *J* = 7.2 Hz, 2H), 7.45 (d, *J* = 7.9 Hz, 1H), 7.36 (t, *J* = 7.2 Hz, 2H), 7.31 (d, *J* = 7.1 Hz, 1H), 7.24 (d, *J* = 3.0 Hz, 1H), 7.22 – 7.17 (m, 1H), 7.14 (d, *J* = 7.5 Hz, 1H), 7.12 – 7.07 (m, 4H), 7.04 (d, *J* = 8.4 Hz, 2H), 7.00 (t, *J* = 7.5 Hz, 1H), 6.77-6.73 (m, 3H), 5.54 (s, 1H), 4.93 (d, *J* = 14.1 Hz, 1H), 4.86 (s, 1H), 4.25 (d, *J* = 14.1 Hz, 1H), 4.22 – 4.13 (m, 1H), 3.19-3.12 (m, 1H), 3.07 – 2.91 (m, 2H), 1.22 (s, 9H). ^{13}C NMR (101 MHz, $CDCl_3$) δ 167.97, 165.57, 151.14, 139.32, 138.00, 136.25, 129.91, 129.33, 128.87, 128.19, 127.26, 126.90, 126.32, 125.23, 122.43, 119.58, 118.35, 111.50, 85.41, 64.60, 47.59, 34.43, 31.24, 22.68, 14.14. HRMS (ESI) m/z calcd for $C_{37}H_{38}N_3O_3^+$ $(M+H)^+$ 572.2913, found 572.2900.

1-(2-(1H-indol-3-yl)ethyl)-4-benzyl-3-hydroxy-3-phenyl-6-(p-tolyl)piperazine-2,5-dione

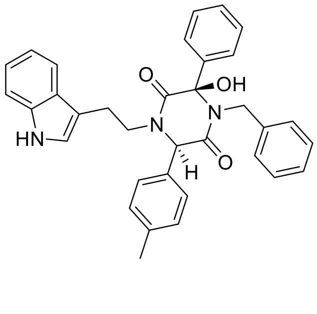

(±) 5i, brown oil, yield 79%, (EA/Hex = 30%, R_f = 0.3), purity 95%. ^{1}H NMR (400 MHz, $CDCl_3$) δ 7.67 (s, 1H), 7.52 (d, *J* = 7.1 Hz, 2H), 7.46 (d, *J* = 7.9 Hz, 1H), 7.38 – 7.27 (m, 4H), 7.24 (d, *J* = 2.6 Hz, 1H), 7.17-7.14 (m, 5H), 7.01 (t, *J* = 7.5 Hz, 1H), 6.86 (d, *J* = 8.0 Hz, 2H), 6.78 (d, *J* = 2.0 Hz, 1H), 6.71 (d, *J* = 8.0 Hz, 2H), 5.44 (s, 1H), 4.91 (d, *J* = 14.1 Hz, 1H), 4.83 (s, 1H), 4.22 (d, *J* = 14.1 Hz, 1H), 4.18-4.11 (m, 1H), 3.20 – 3.13 (m, 1H), 3.05 – 2.90 (m, 2H), 2.24 (s, 3H). ^{13}C NMR (101 MHz, $CDCl_3$) δ 167.84, 165.36, 139.46, 138.03, 136.22, 130.09, 129.34, 128.93, 127.24, 126.76, 122.33, 119.62, 118.35, 111.58, 85.38, 64.68, 47.63, 22.61, 20.95. HRMS (ESI) m/z calcd for $C_{34}H_{32}N_3O_3^+$ $(M+H)^+$ 530.2444, found 530.2456.

1-(2-(1H-indol-3-yl)ethyl)-6-(4-bromophenyl)-3-hydroxy-4-(4-methoxybenzyl)-3-phenylpiperazine-2,5-dione

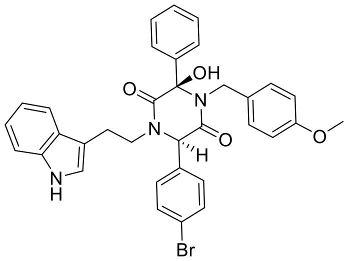

(±) 5j, brown oil, yield 71%, (EA/Hex = 30%, R_f = 0.25), purity 97%. ^{1}H NMR (400 MHz, $CDCl_3$) δ 7.84 (s, 1H), 7.48 – 7.42 (m, 3H), 7.28 (s, 1H), 7.23 (s, 1H), 7.13 (dt, *J* = 17.2, 6.3 Hz, 7H), 7.00 (t, *J* = 7.4 Hz, 1H), 6.86 (d, *J* = 8.1 Hz, 2H), 6.72 (s, 1H), 6.67 (d, *J* = 8.0 Hz, 2H), 5.62 (s, 1H), 4.84 (d, *J* = 14.0 Hz, 1H), 4.79 (s, 1H), 4.21 (d, *J* = 14.0 Hz, 1H), 4.12 (ddd, *J* = 12.8, 7.9, 4.8 Hz, 1H), 3.79 (s, 3H), 3.16 – 3.07 (m, 1H), 3.04 – 2.96 (m, 1H), 2.89 (dd, *J* = 13.3, 7.5 Hz, 1H). ^{13}C NMR (101 MHz, $CDCl_3$) δ 167.89, 164.81, 158.89, 139.36, 136.25, 132.29, 131.36, 130.86, 129.86, 129.21, 128.51, 126.83, 126.28, 122.58, 122.19, 119.66, 118.29, 113.55, 111.43, 85.41, 64.39, 55.35, 47.42, 47.20, 22.71. HRMS (ESI) m/z calcd for $C_{34}H_{31}BrN_3O_4^+$ $(M+H)^+$ 624.1493, found 624.1472.

1-(2-(1H-indol-3-yl)ethyl)-6-(3,4-dichlorophenyl)-3-hydroxy-4-(4-methoxybenzyl)-3-phenylpiperazine-2,5-dione

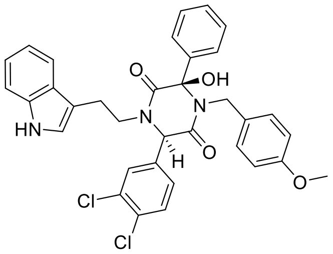

(±) 5k, brown oil, yield 66%, (EA/Hex = 30%, R_f = 0.25), purity 95%. ^{1}H NMR (400 MHz, $CDCl_3$) δ 7.75 (s, 1H), 7.46 (dd, *J* = 7.8, 5.7 Hz, 3H), 7.28 (d, *J* = 4.1 Hz, 1H), 7.24 (s, 1H), 7.20 – 7.14 (m, 3H), 7.09 (t, *J* = 7.5 Hz, 3H), 7.02 (t, *J* = 7.4 Hz, 1H), 6.89 (d, *J* = 8.6 Hz, 2H), 6.75 – 6.71 (m, 2H), 6.68 (dd, *J* = 8.4, 1.8 Hz, 1H), 5.59 (s, 1H), 4.87 (d, *J* = 13.9 Hz, 1H), 4.70 (s, 1H), 4.26 (d, *J* = 13.9 Hz, 1H), 4.20 – 4.12 (m, 1H), 3.81 (s, 3H), 3.19 – 3.10 (m, 1H), 3.05 – 2.97 (m, 1H), 2.89 (dd, *J* = 13.4, 7.7 Hz, 1H). ^{13}C NMR (101 MHz, $CDCl_3$) δ 167.97, 164.46, 158.95, 139.20, 136.24, 133.33, 132.51, 130.92, 130.08, 129.77, 129.48, 128.59, 126.72, 126.08, 122.42, 119.72, 118.23, 113.55, 111.52, 111.23, 85.23, 64.01, 55.36, 47.47, 47.13, 22.70. HRMS (ESI) m/z calcd for $C_{34}H_{30}Cl_2N_3O_4^+$ $(M+H)^+$ 614.1608, found 614.1529.

1-(2-(1H-indol-3-yl)ethyl)-6-(4-fluorophenyl)-3-hydroxy-4-(4-methoxybenzyl)-3-phenylpiperazine-2,5-dione

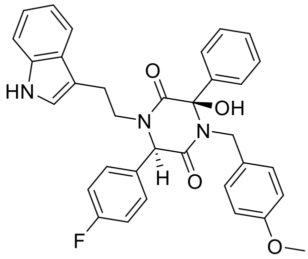

(±) 5l, brown oil, yield 70%, (EA/Hex = 30%, R_f = 0.25), purity 93%. ^{1}H NMR (400 MHz, $CDCl_3$) δ 7.82 (s, 1H), 7.46 (dd, *J* = 7.8, 4.8 Hz, 3H), 7.27 – 7.23 (m, 2H), 7.18 – 7.10 (m, 5H), 7.01 (t, *J* = 7.5 Hz, 1H), 6.87 (d, *J* = 8.6 Hz, 2H), 6.80 – 6.70 (m, 5H), 5.60 (s, 1H), 4.85 (d, *J* = 16.1 Hz, 2H), 4.21 (d, *J* = 14.0 Hz, 1H), 4.13 (ddd, *J* = 13.1, 8.3, 4.6 Hz, 1H), 3.80 (s, 3H), 3.18 – 3.08 (m, 1H), 3.05 – 2.97 (m, 1H), 2.91 (dd, *J* = 13.2, 7.7 Hz, 1H). ^{13}C NMR (101 MHz, $CDCl_3$) δ 167.89, 165.15, 158.88, 139.41,

136.24, 130.85, 129.91, 129.17, 128.51, 126.85, 126.30, 122.35, 119.64, 118.30, 115.30, 115.09, 113.54, 111.45, 85.38, 64.24, 55.34, 47.28, 47.14, 46.93, 22.70. HRMS (ESI) m/z calcd for $C_{34}H_{31}FN_3O_4^+$ $(M+H)^+$ 614.1529, found 614.1522.

1-(2-(1H-indol-3-yl)ethyl)-3-hydroxy-4-(4-methoxybenzyl)-6-(4-nitrophenyl)-3-phenylpiperazine-2,5-dione

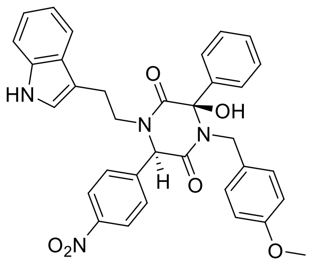

(±) 5m, brown oil, yield 64%, (EA/Hex = 30%, R_f = 0.25), purity 91%. ^{1}H NMR (400 MHz, $CDCl_3$) δ 7.82 (d, *J* = 8.8 Hz, 2H), 7.71 (s, 1H), 7.46 (dd, *J* = 14.1, 8.2 Hz, 3H), 7.30 (d, *J* = 8.2 Hz, 1H), 7.22 (d, *J* = 7.3 Hz, 1H), 7.17 (dd, *J* = 11.2, 4.0 Hz, 1H), 7.09 (t, *J* = 7.8 Hz, 2H), 7.05 – 6.98 (m, 3H), 6.91 (dd, *J* = 8.8, 2.2 Hz, 4H), 6.71 (d, *J* = 2.2 Hz, 1H), 5.58 (s, 1H), 4.87 (d, *J* = 13.9 Hz, 1H), 4.80 (s, 1H), 4.29 (d, *J* = 13.9 Hz, 1H), 4.26 – 4.17 (m, 1H), 3.83 (s, 3H), 3.18 (dt, *J* = 15.5, 7.9 Hz, 1H), 3.09 – 2.99 (m, 1H), 2.94 – 2.85 (m, 1H). ^{13}C NMR (101 MHz, $CDCl_3$) δ 168.05, 164.10, 159.02, 147.54, 140.25, 139.30, 136.27, 130.96, 129.66, 129.37, 128.59, 127.40, 126.65, 125.99, 123.12, 122.48, 119.75, 118.13, 113.56, 111.56, 111.10, 85.12, 64.72, 55.36, 47.62, 47.07, 29.71, 22.72. HRMS (ESI) m/z calcd for $C_{34}H_{31}N_4O_6^+$ $(M+H)^+$ 591.2293, found 591.2290.

1-(2-(1H-indol-3-yl)ethyl)-3-hydroxy-4-(4-methoxybenzyl)-3,6-diphenylpiperazine-2,5-dione

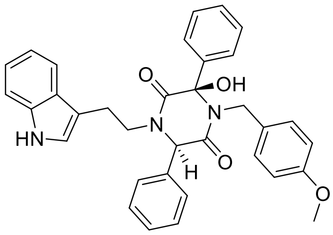

(±) 5n, brown oil, yield 73%, (EA/Hex = 30%, R_f = 0.3), purity 92%. ^{1}H NMR (400 MHz, $CDCl_3$) δ 7.75 (s, 1H), 7.48 (t, *J* = 7.0 Hz, 3H), 7.29 (d, *J* = 8.1 Hz, 1H), 7.25 – 7.21 (m, 1H), 7.18 – 7.11 (m, 6H), 7.07-7.00 (m, 3H), 6.90-6.84 (m, 4H), 6.78 (d, *J* = 1.3 Hz, 1H), 5.49 (s, 1H), 4.92 – 4.83 (m, 2H), 4.22 – 4.12 (m, 2H), 3.82 (s, 3H), 3.20 – 3.10 (m, 1H), 3.07 – 2.99 (m, 1H), 2.97 – 2.88 (m, 1H). ^{13}C NMR (101 MHz, $CDCl_3$) δ 167.91, 165.20, 158.84, 139.37, 136.20, 133.06, 130.89, 130.01, 129.02, 128.40, 128.26, 126.76, 126.34, 122.30, 119.62, 118.36, 113.48, 111.57, 111.38, 85.35, 64.78, 55.34, 47.44, 47.16, 22.64. HRMS (ESI) m/z calcd for $C_{34}H_{32}N_3O_4^+$ $(M+H)^+$ 546.2393, found 546.2388.

1-(2-(1H-indol-3-yl)ethyl)-3-(4-bromophenyl)-3-hydroxy-4-(4-methoxybenzyl)-6-phenylpiperazine-2,5-dione

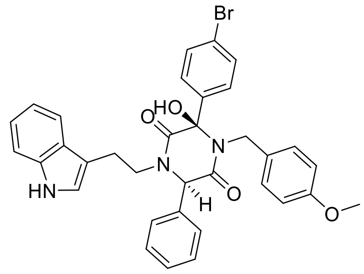

(±) 5o, brown oil, yield 78% (EA/Hex = 30%, R_f = 0.25), purity 94%. ^{1}H NMR (400 MHz, $CDCl_3$) δ 7.92 (s, 1H), 7.45 – 7.40 (m, 3H), 7.21 (d, *J* = 5.9 Hz, 4H), 7.11 (d, *J* = 7.9 Hz, 3H), 6.98 (t, *J* = 8.2 Hz, 3H), 6.85 (dd, *J* = 15.6, 8.1 Hz, 4H), 6.72 (s, 1H), 5.63 (s, 1H), 4.93 (s, 1H), 4.80 (d, *J* = 14.0 Hz, 1H), 4.16 (dd, *J* = 15.5, 8.5 Hz, 2H), 3.77 (s, 3H), 3.09 (dd, *J* = 14.1, 7.0 Hz, 1H), 3.01 – 2.90 (m, 2H). ^{13}C NMR (101 MHz, $CDCl_3$) δ 167.52, 165.16, 158.90, 138.47, 136.26, 133.09, 131.44, 130.81, 129.78, 128.72, 128.18, 126.92, 126.68, 123.44, 122.33, 119.62, 118.34, 113.58, 111.46, 85.25, 64.67, 55.35, 47.61, 47.02, 22.73. HRMS (ESI) m/z calcd for $C_{34}H_{31}BrN_3O_4^+$ $(M+H)^+$ 624.1487, found 624.1493.

1-(2-(1H-indol-3-yl)ethyl)-3-(furan-2-yl)-3-hydroxy-4-(4-methoxybenzyl)-6-phenylpiperazine-2,5-dione

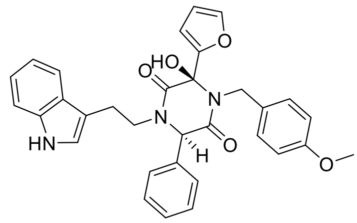

(±) 5p, brown oil, yield 70%, (EA/Hex = 30%, R_f = 0.25), purity 95%. ^{1}H NMR (400 MHz, $CDCl_3$) δ 7.89 (s, 1H), 7.45 (d, *J* = 7.9 Hz, 1H), 7.39 (d, *J* = 8.5 Hz, 2H), 7.25 – 7.17 (m, 5H), 7.13 (t, *J* = 7.6 Hz, 1H), 7.07 – 6.98 (m, 3H), 6.83 (d, *J* = 8.5 Hz, 2H), 6.77 (d, *J* = 1.5 Hz, 1H), 6.20 – 6.10 (m, 2H), 5.49 (s, 1H), 4.87 (s, 1H), 4.70 (d, *J* = 14.2 Hz, 1H), 4.34 (d, *J* = 14.1 Hz, 1H), 4.15 (t, *J* = 8.9 Hz, 1H), 3.78 (s, 3H), 3.11 (dd, *J* = 19.4, 9.8 Hz, 1H), 3.04 – 2.95 (m, 2H). ^{13}C NMR (101 MHz, $CDCl_3$) δ 165.76, 164.96, 158.79, 151.35, 143.08, 136.26, 134.04, 130.69, 129.83, 128.42, 126.98, 122.28, 119.57, 118.41, 113.50, 111.50, 110.92, 109.93, 81.34, 65.00, 55.31, 47.42, 46.64, 22.68. HRMS (ESI) m/z calcd for $C_{32}H_{30}N_3O_5^+$(M+H)$^+$ 536.2177, found 536.2180

NMR Characterization Figures of Products

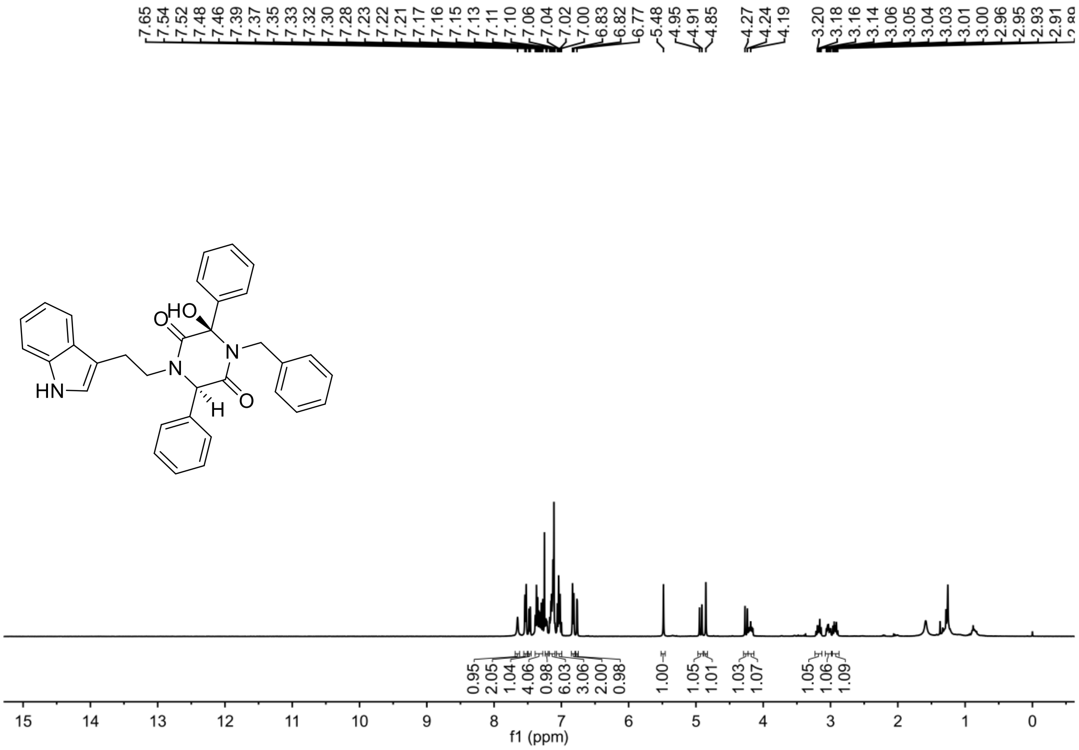

^{1}H NMR spectrum of **(±) 5a**

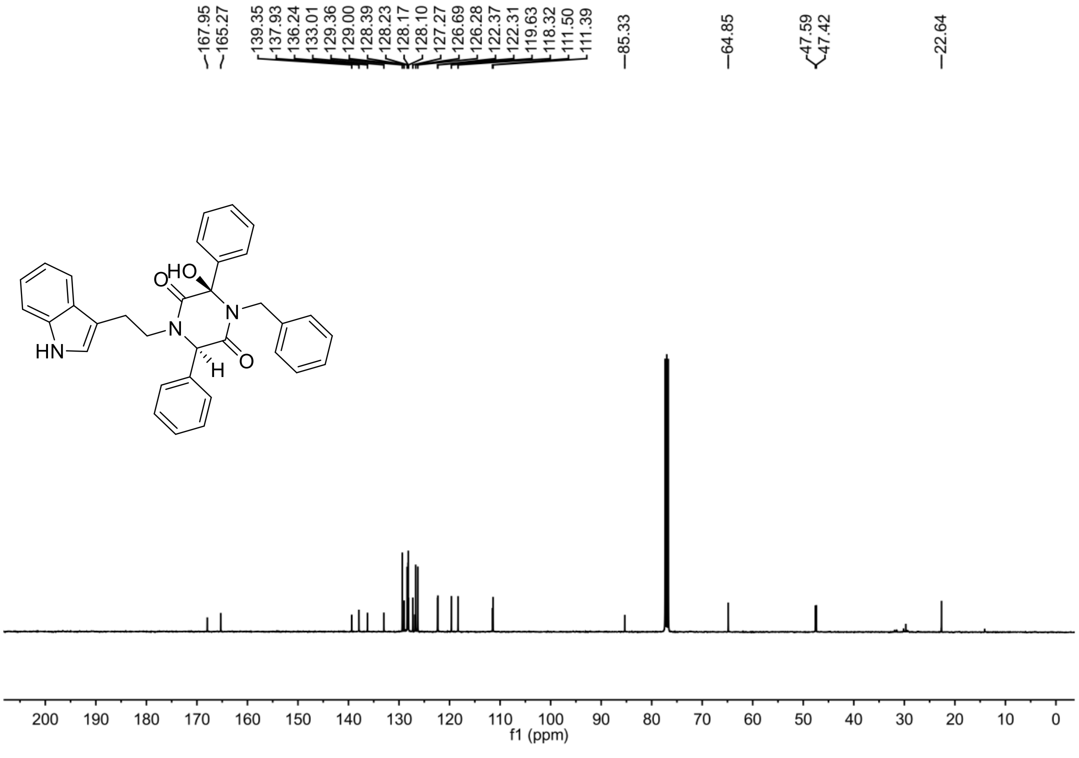

^{13}C NMR spectrum of **(±) 5a**

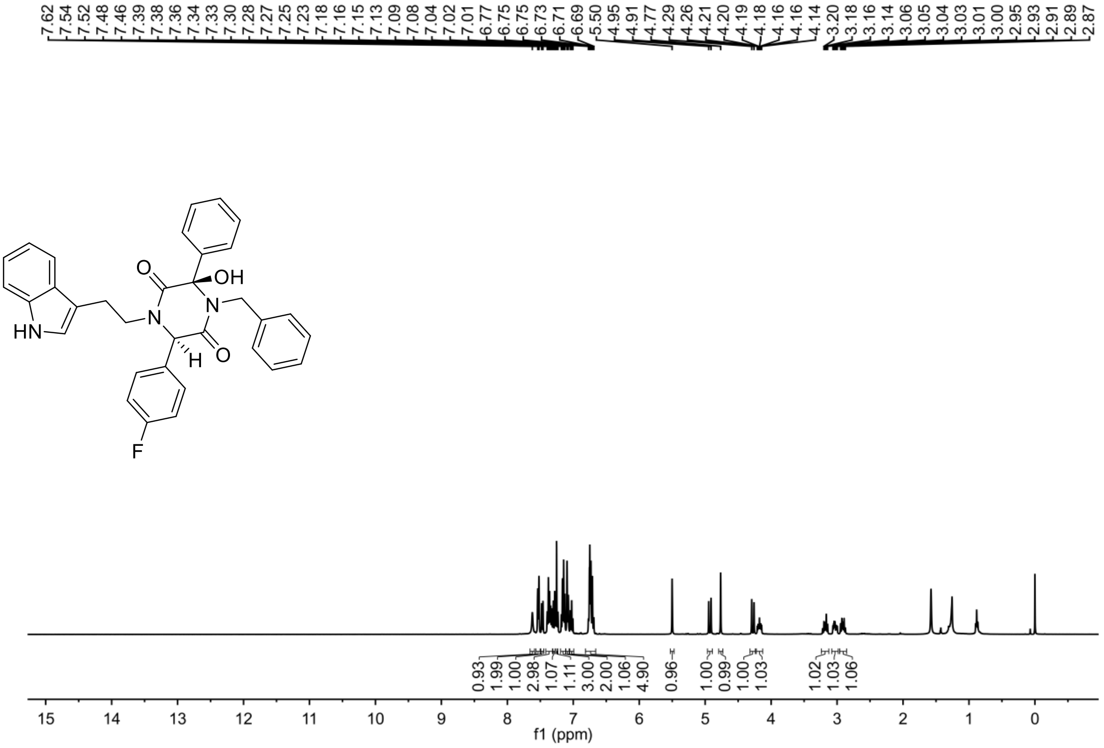

^{1}H NMR spectrum of **(±) 5b**

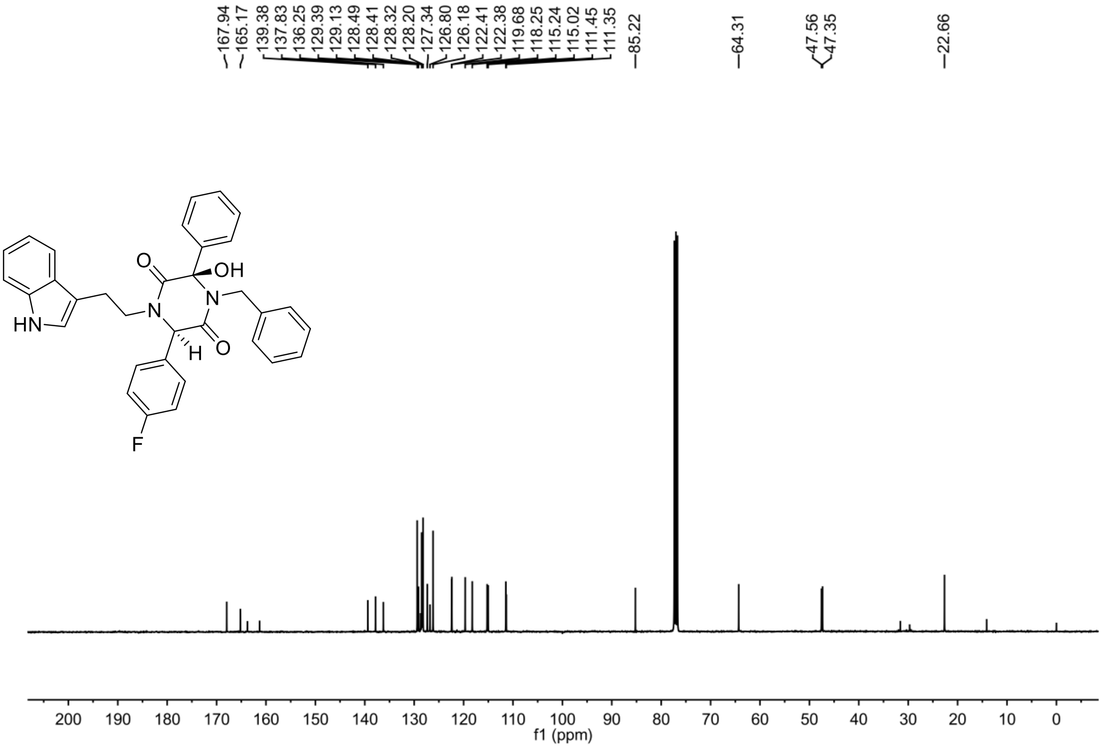

^{13}C NMR spectrum of **(±) 5b**

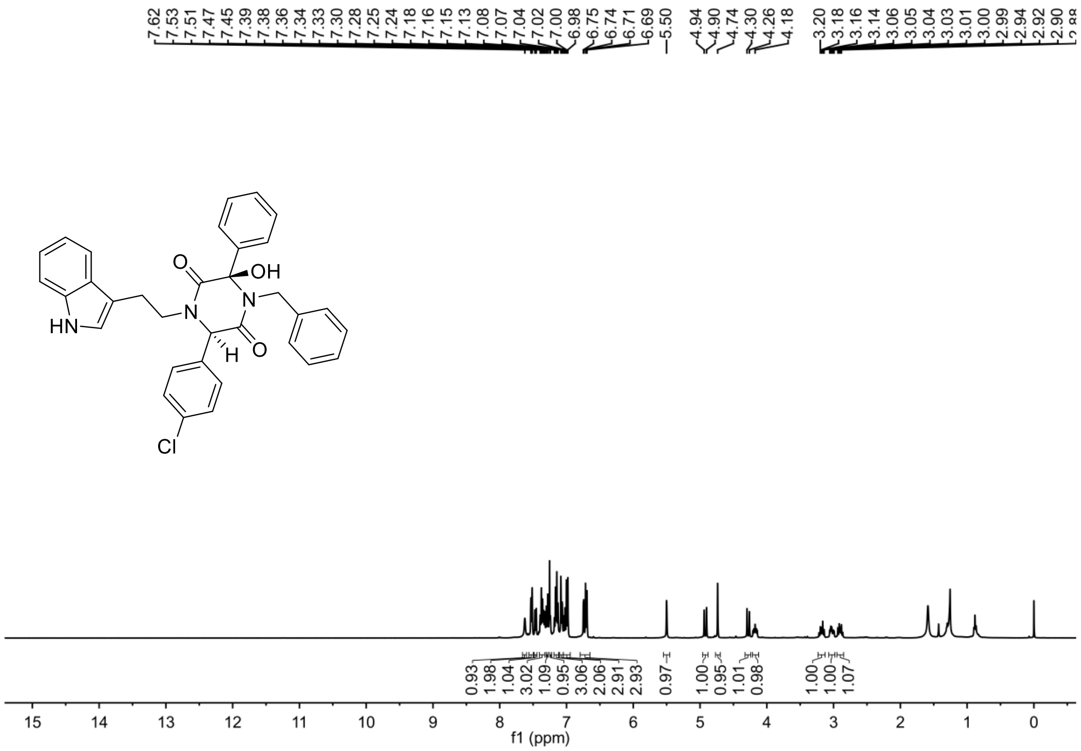

^{1}H NMR spectrum of **(±) 5c**

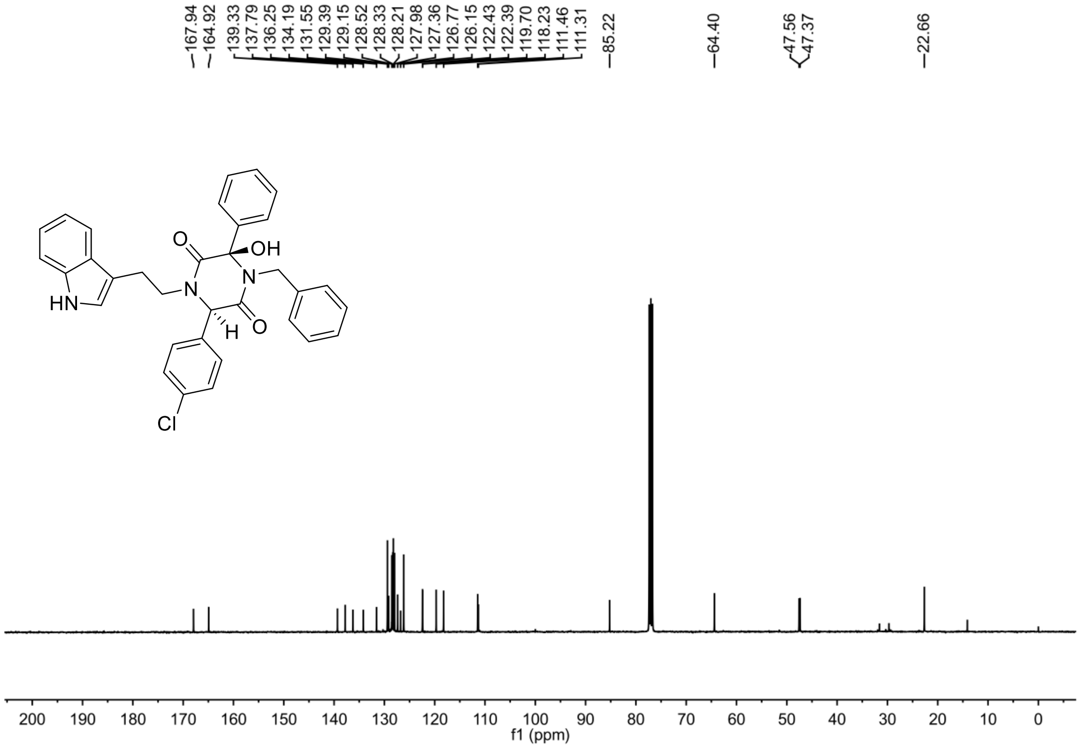

^{13}C NMR spectrum of **(±) 5c**

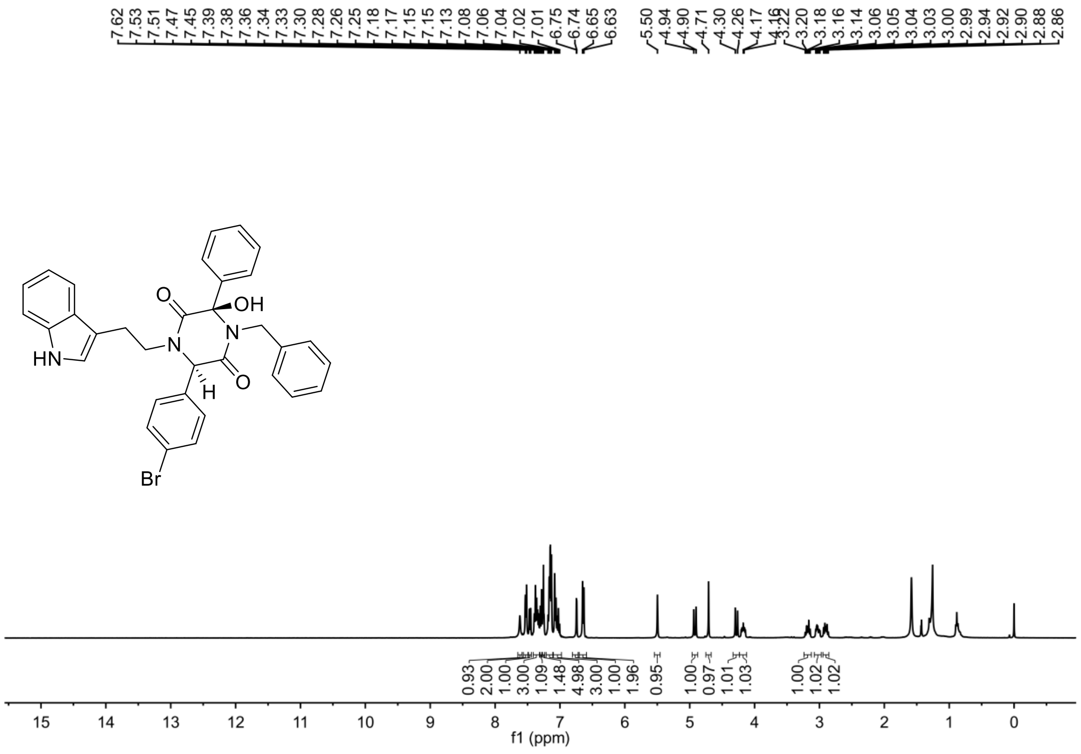

^{1}H NMR spectrum of **(±) 5d**

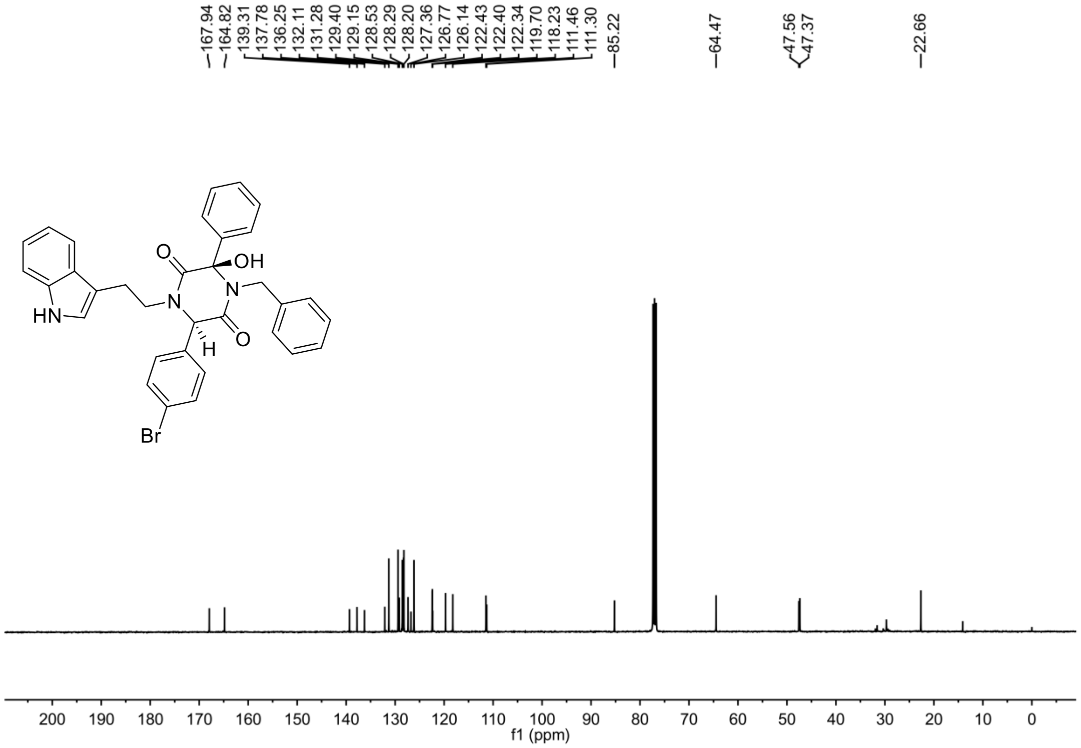

^{13}C NMR spectrum of **(±) 5d**

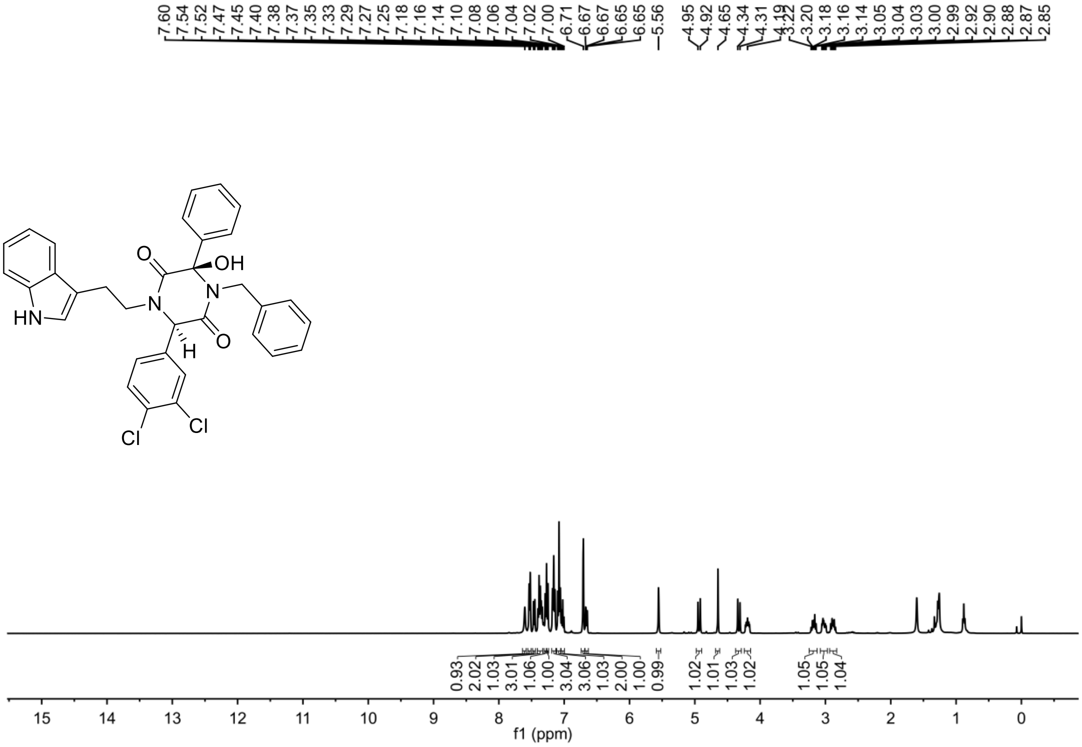

^{1}H NMR spectrum of **(±) 5e**

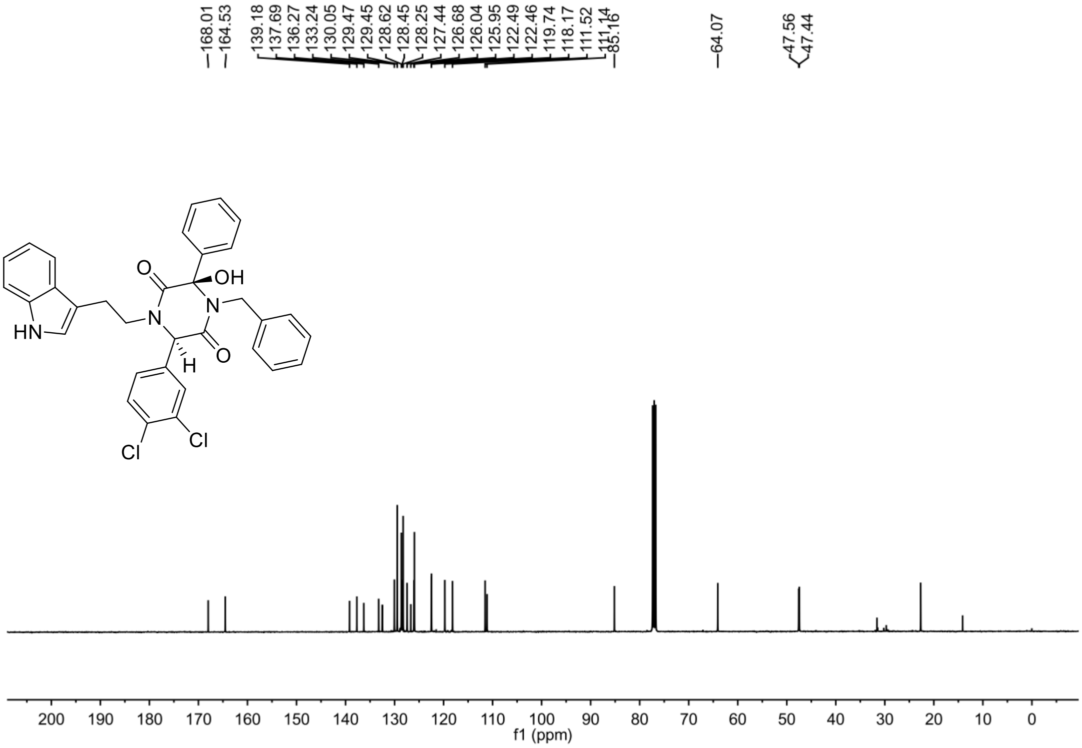

^{13}C NMR spectrum of **(±) 5e**

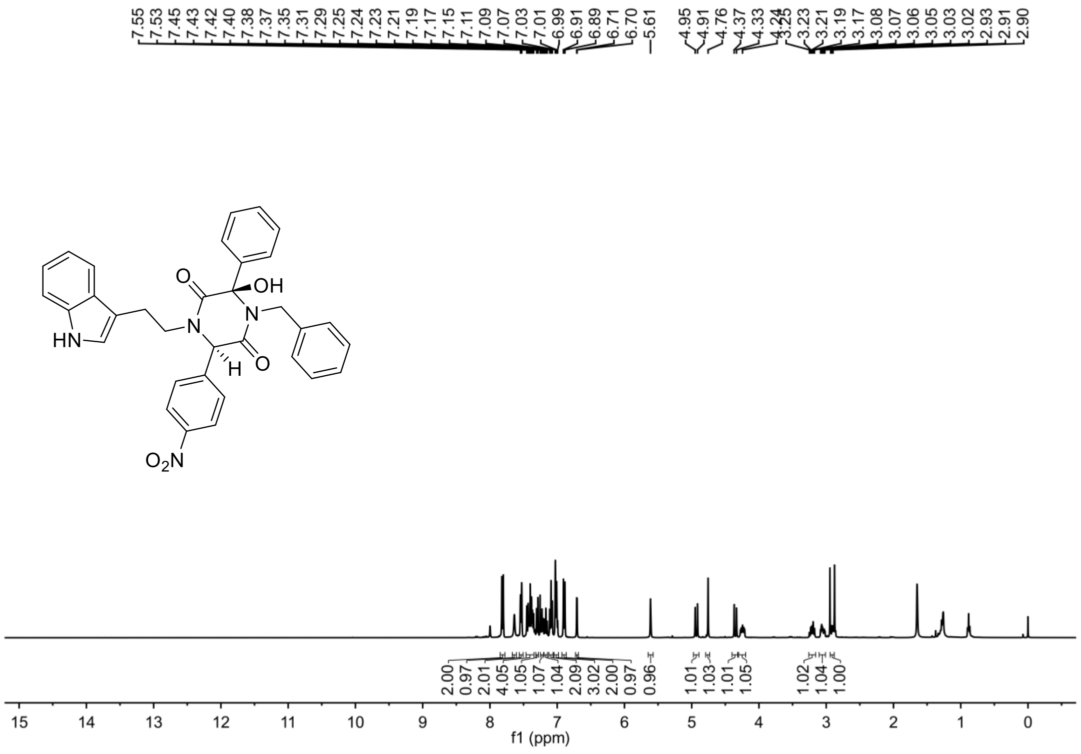

^{1}H NMR spectrum of **(±) 5f**

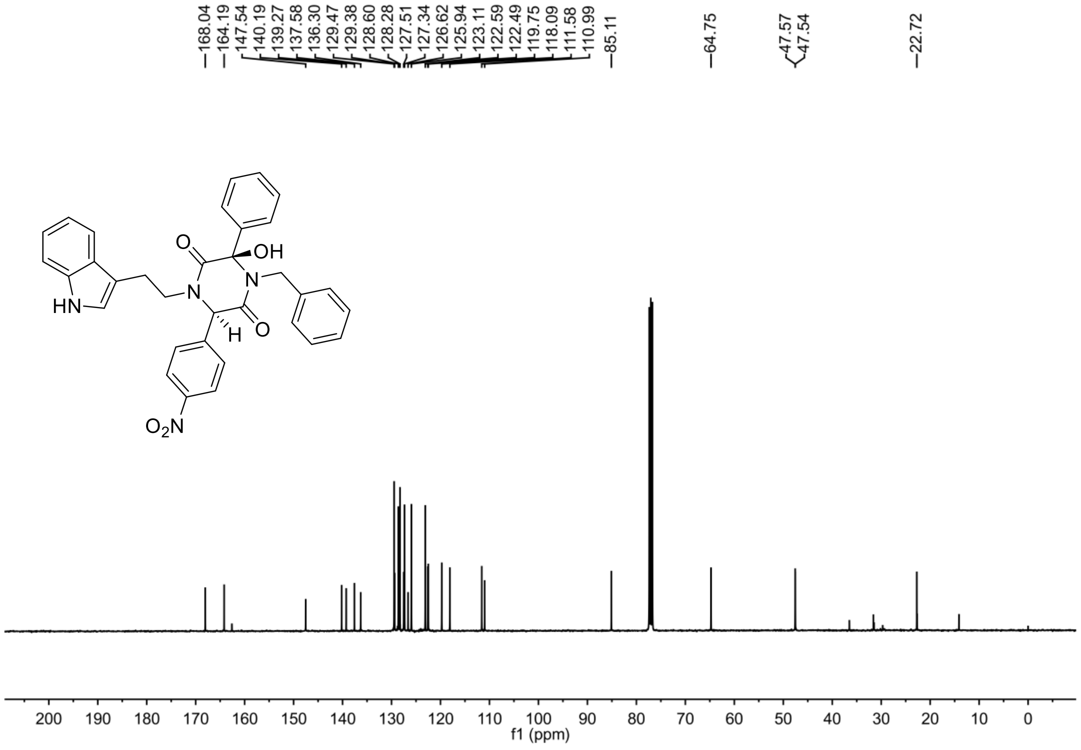

^{13}C NMR spectrum of **(±) 5f**

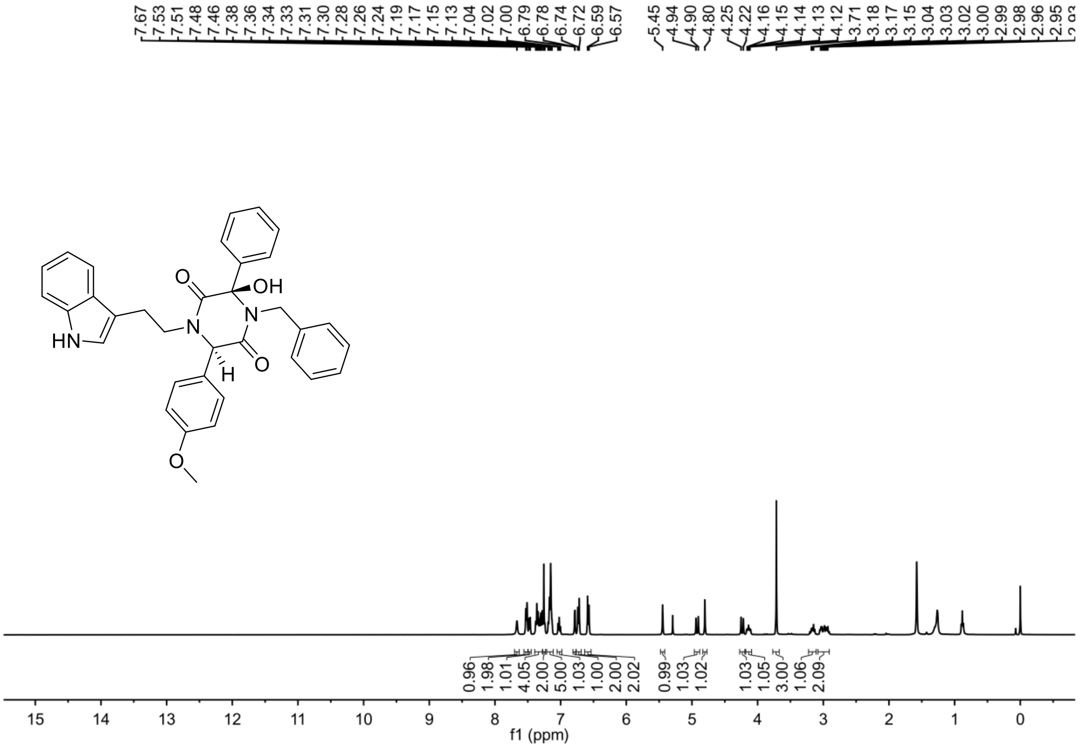

^{1}H NMR spectrum of **(±) 5g**

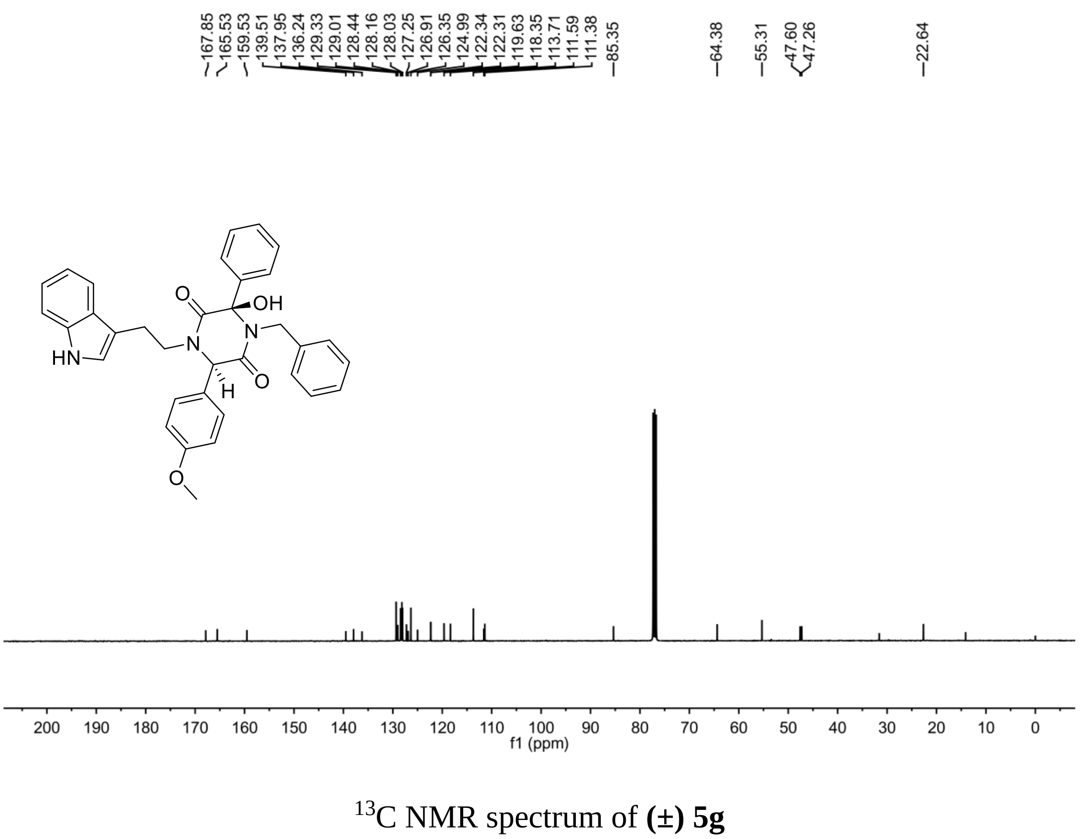

^{13}C NMR spectrum of **(±) 5g**

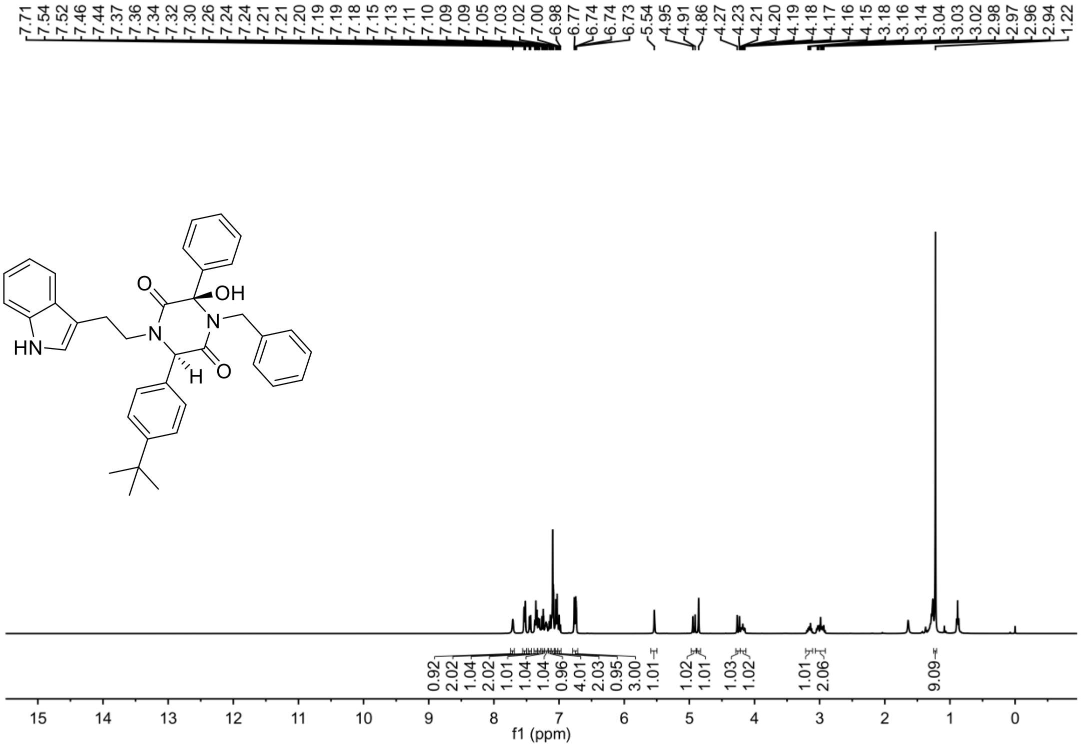

^{1}H NMR spectrum of **(±) 5h**

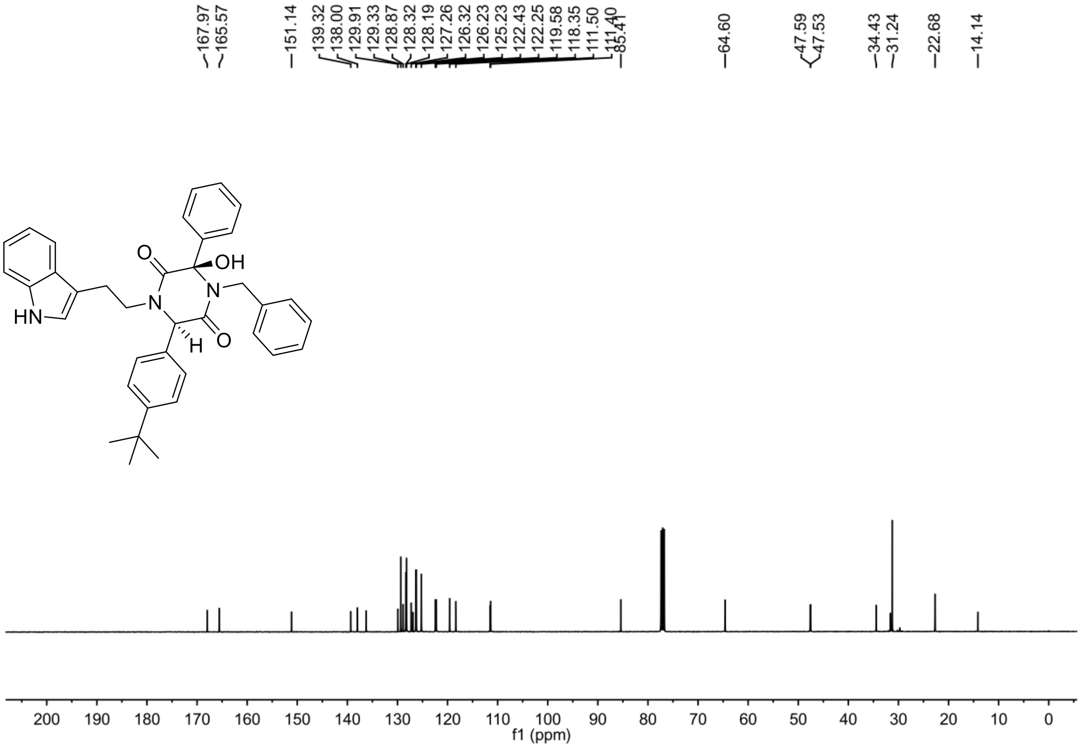

^{13}C NMR spectrum of **(±) 5h**

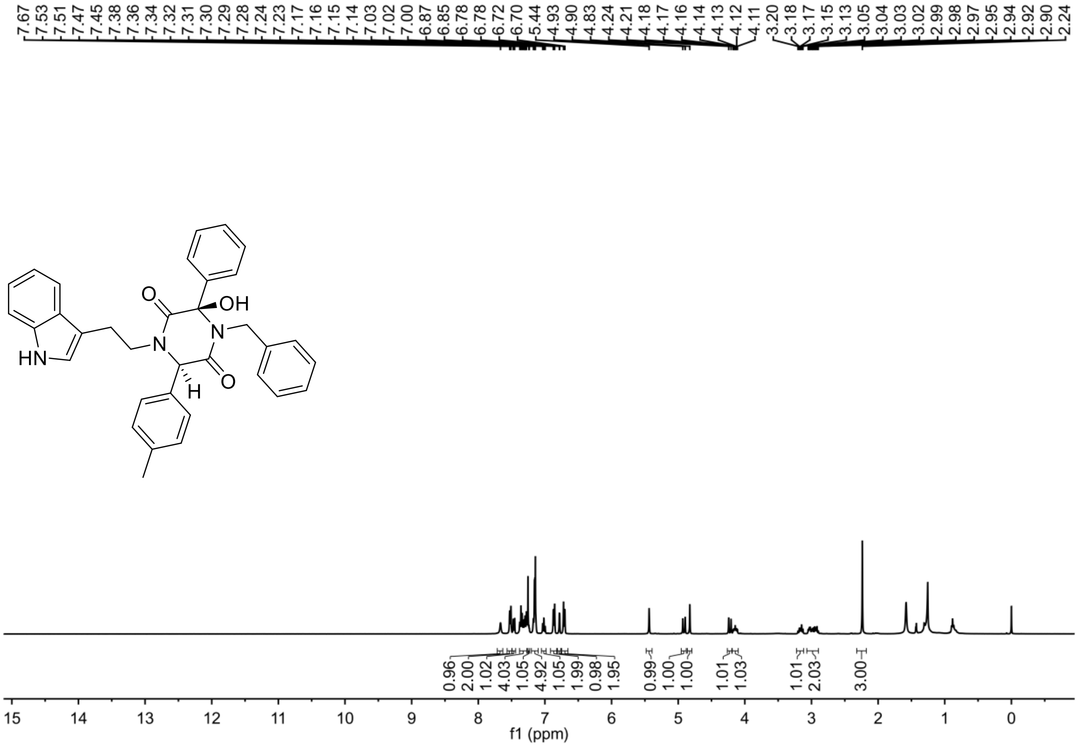

^{1}H NMR spectrum of **(±) 5i**

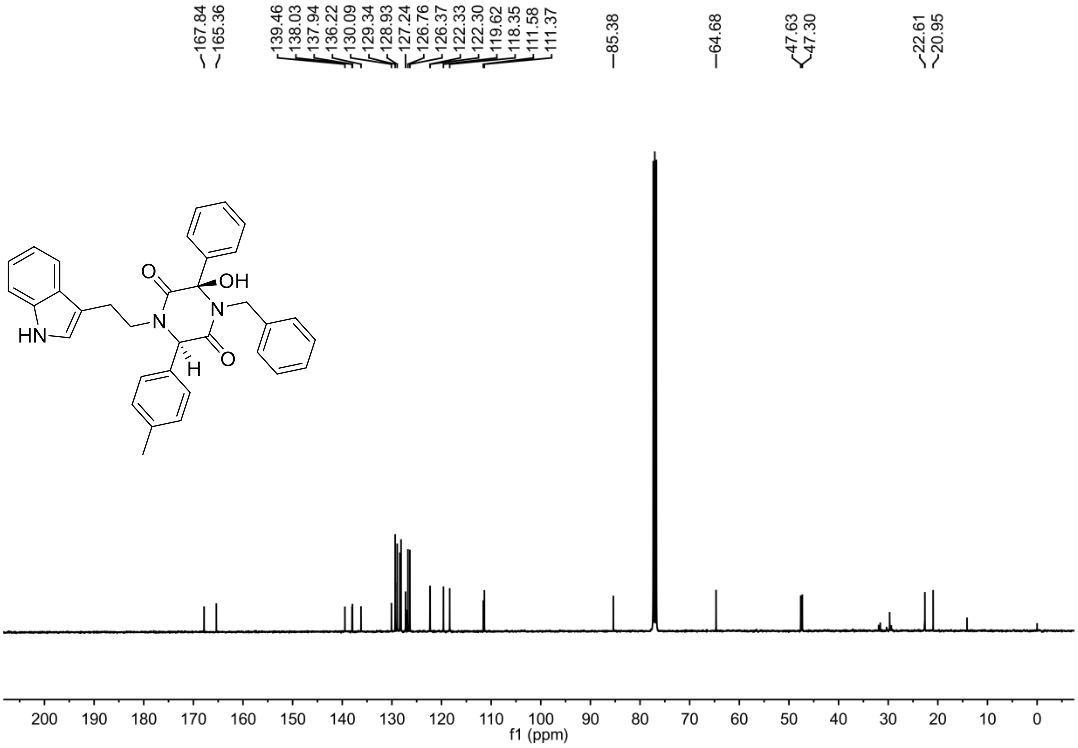

^{13}C NMR spectrum of **(±) 5i**

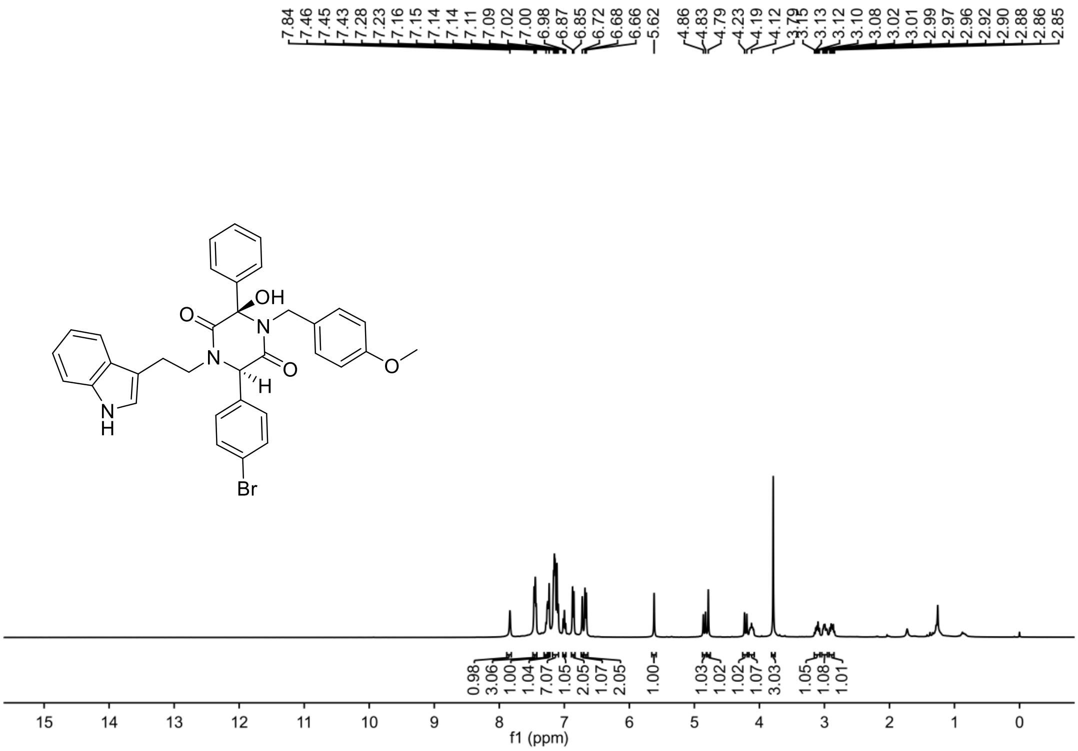

^{1}H NMR spectrum of **(±) 5j**

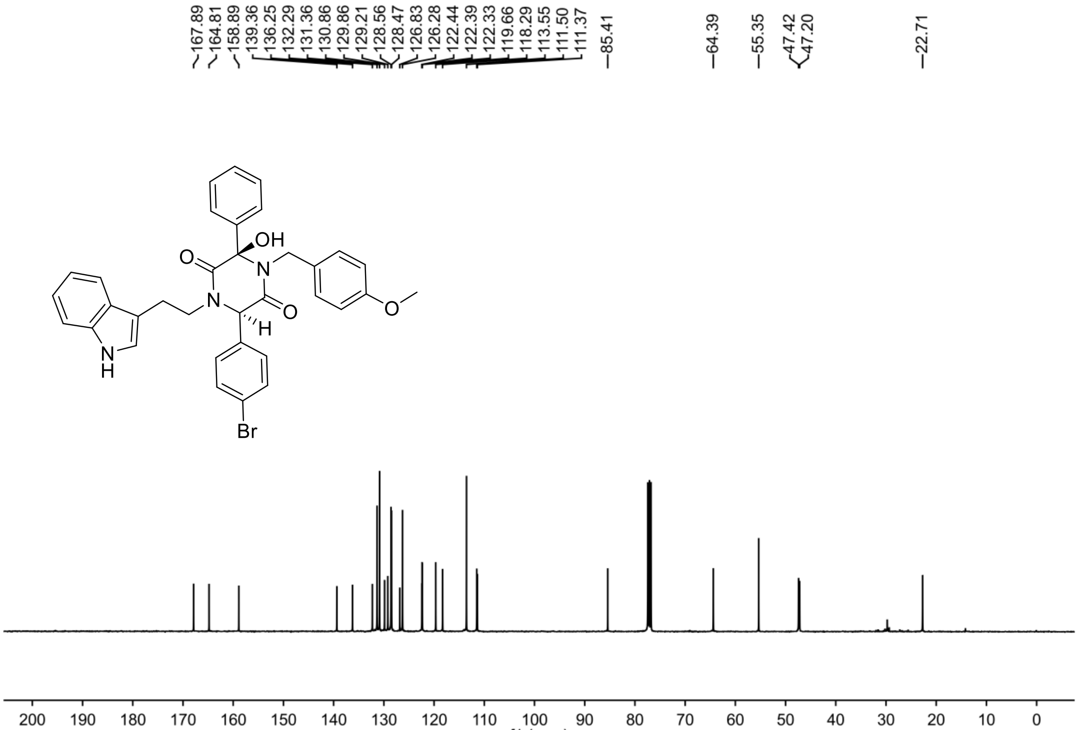

^{13}C NMR spectrum of **(±) 5j**

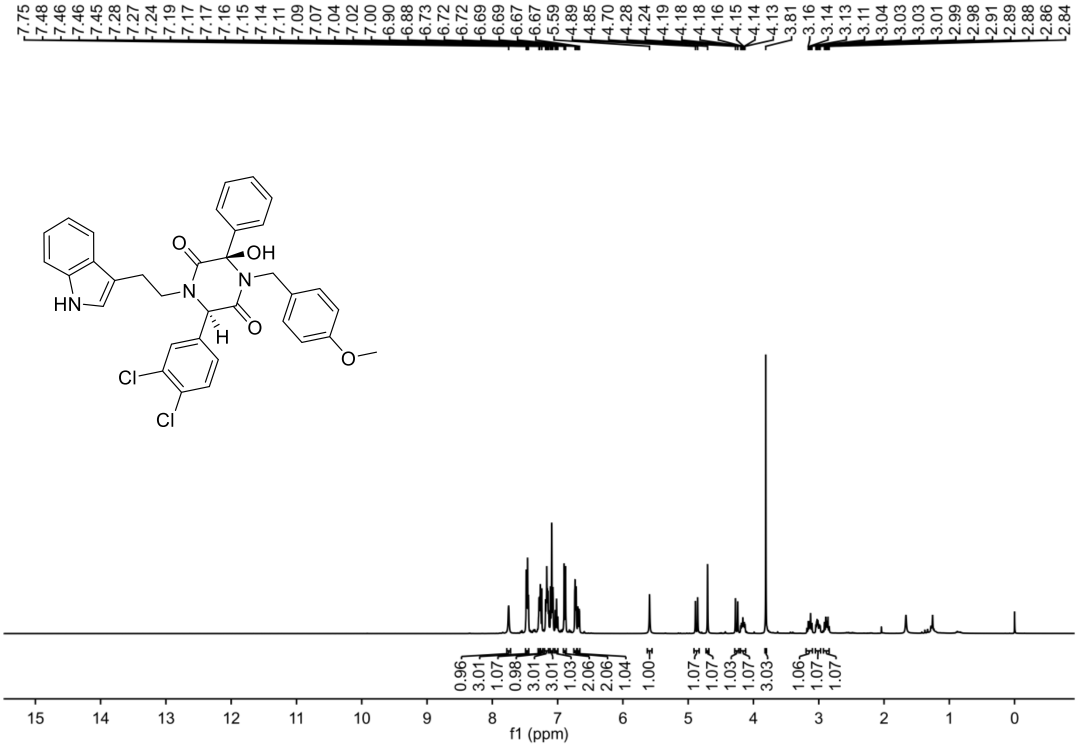

^{1}H NMR spectrum of **(±) 5k**

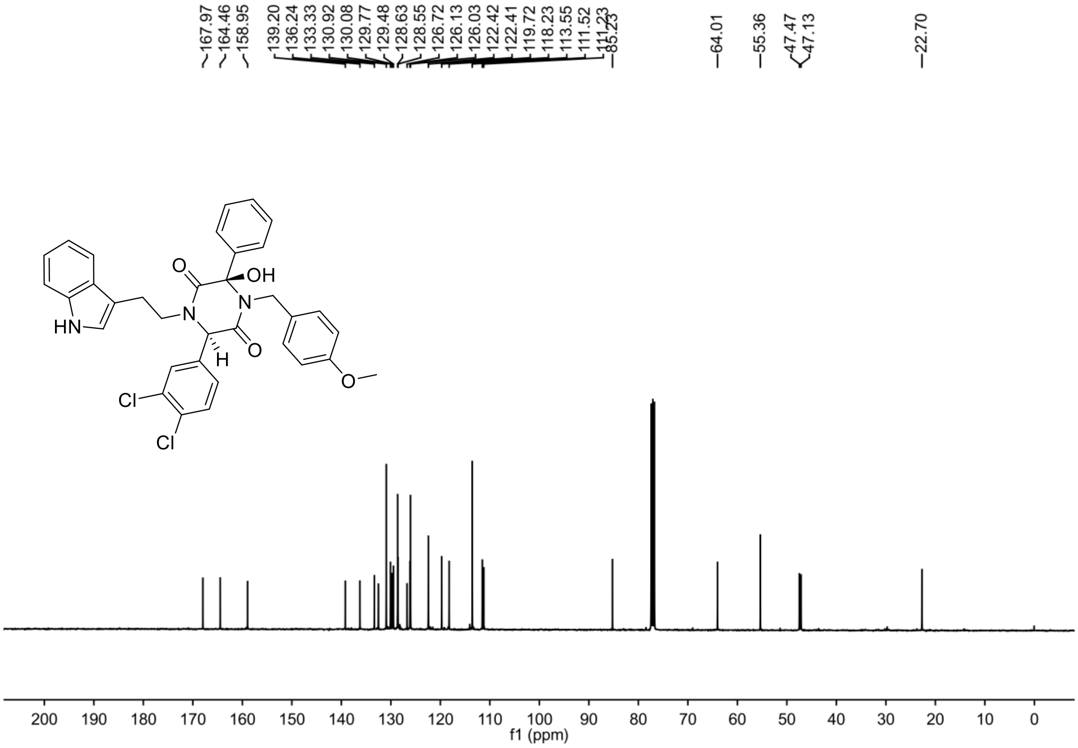

^{13}C NMR spectrum of **(±) 5k**

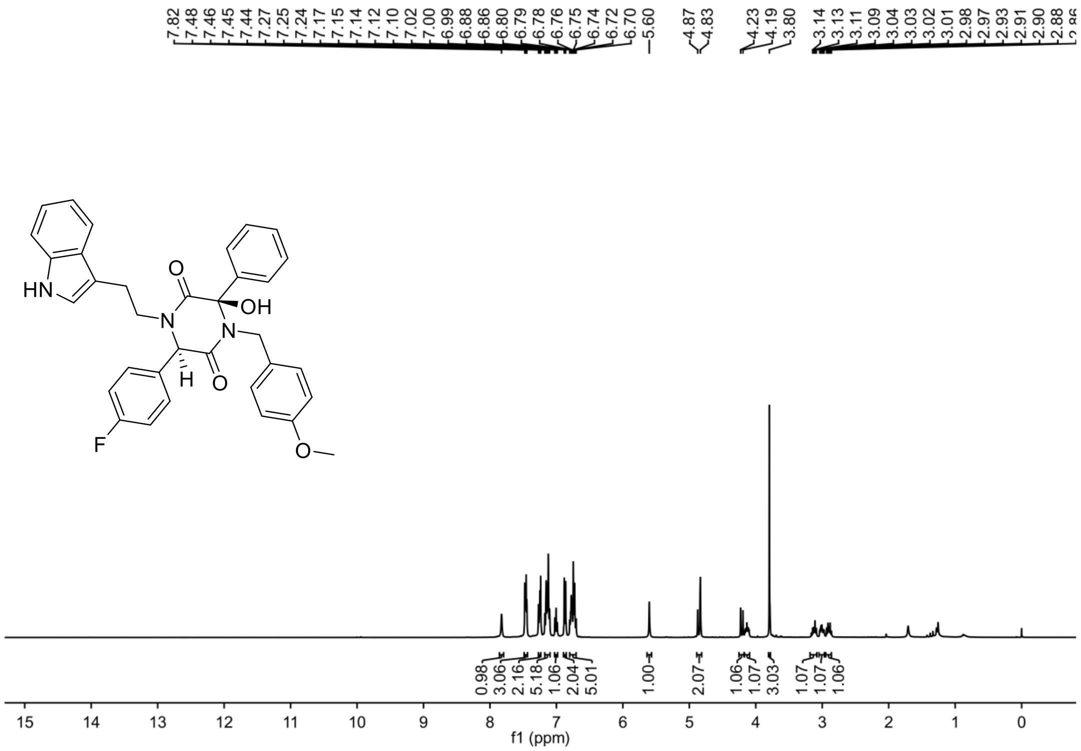

^{1}H NMR spectrum of **(±) 5l**

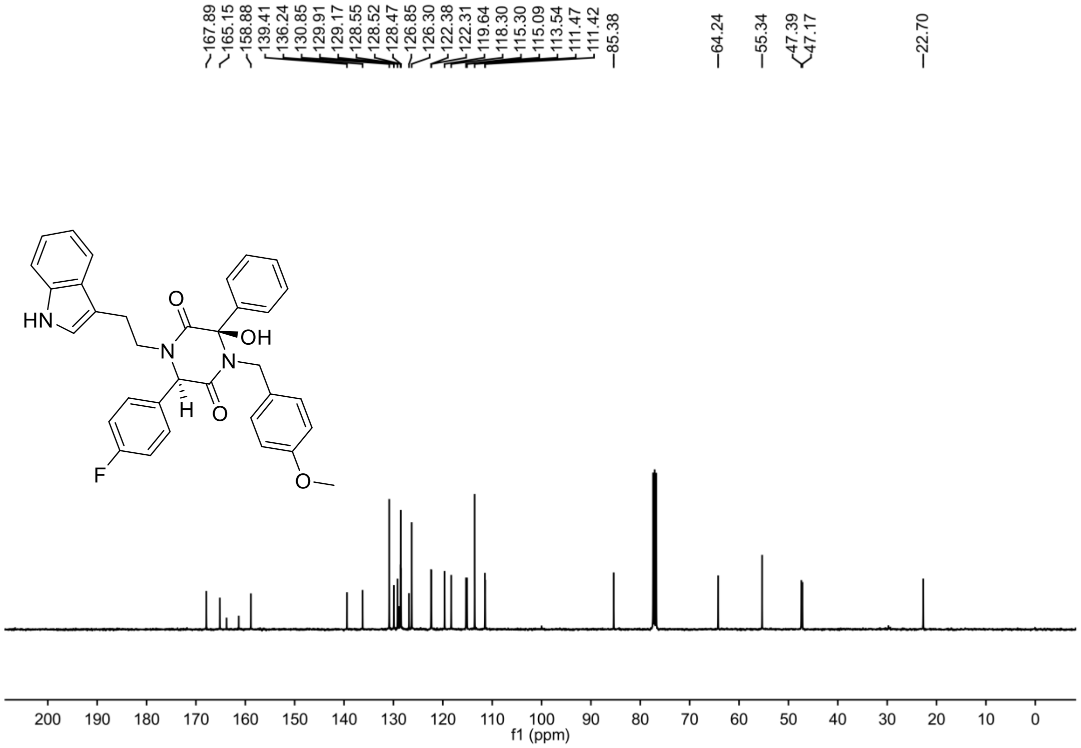

^{13}C NMR spectrum of **(±) 5l**

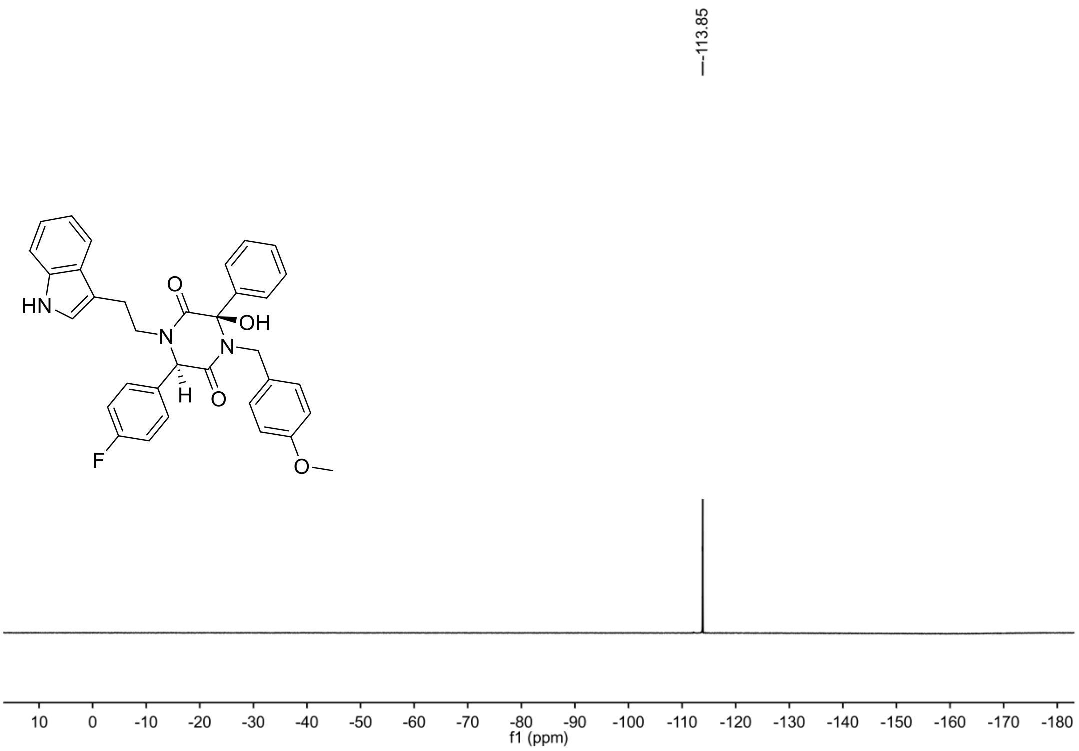

^{19}F NMR spectrum of **(±) 5l**

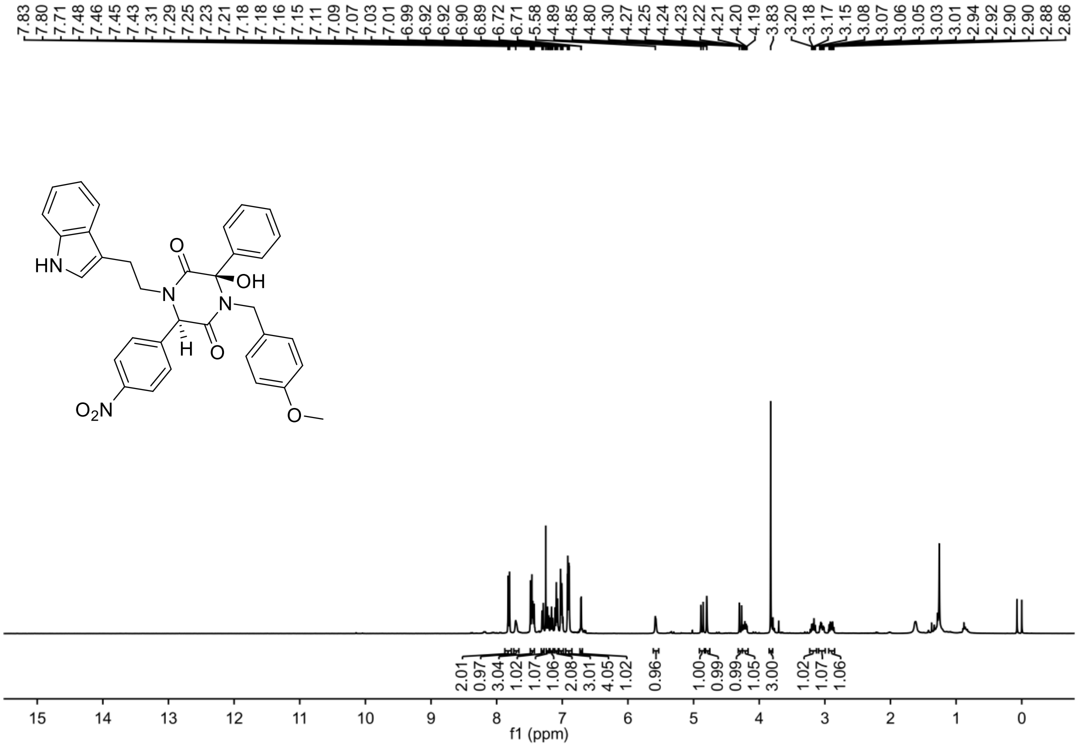

^{1}H NMR spectrum of **(±) 5m**

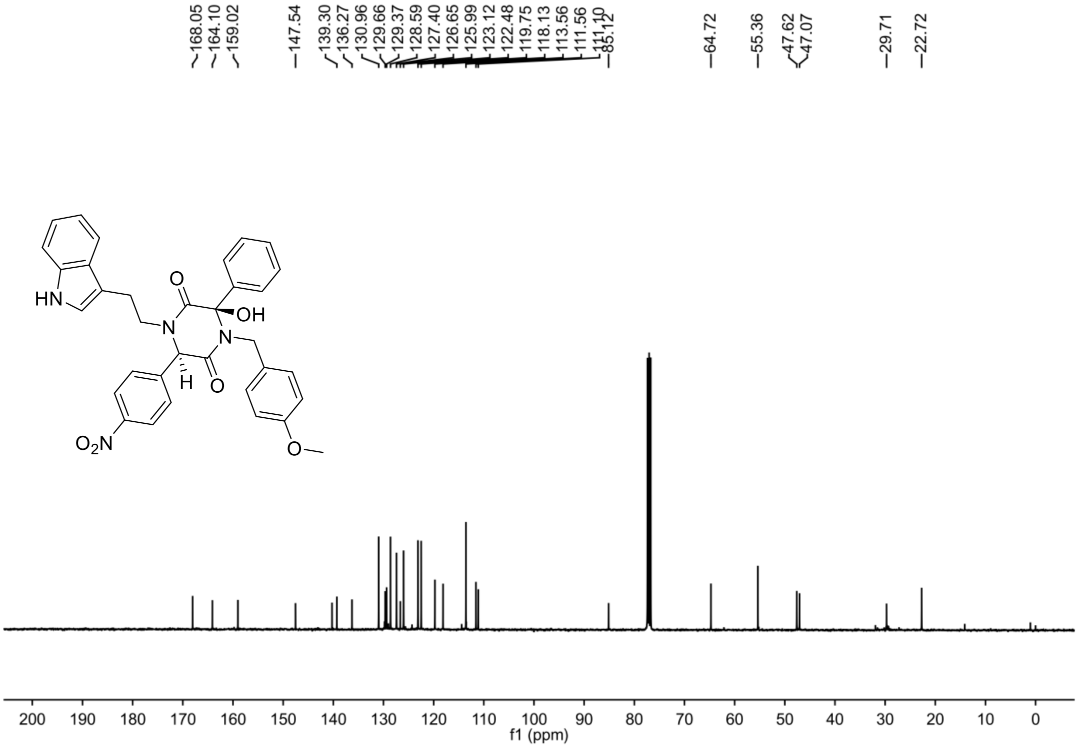

^{13}C NMR spectrum of **(±) 5m**

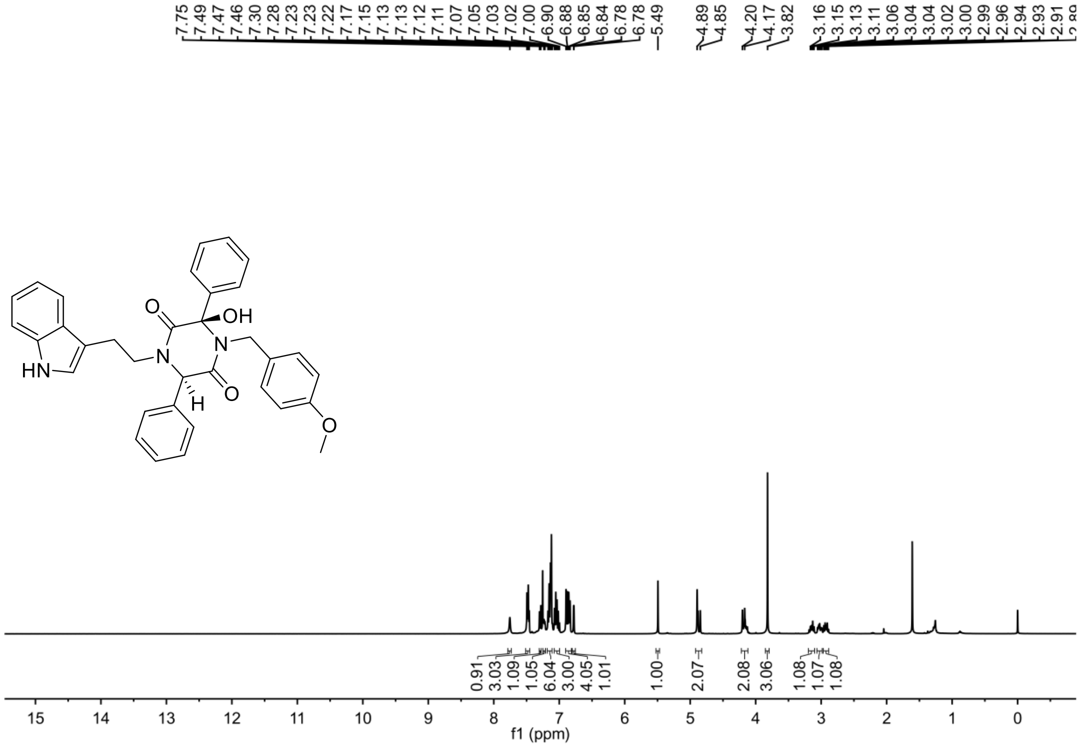

^{1}H NMR spectrum of **(±) 5n**

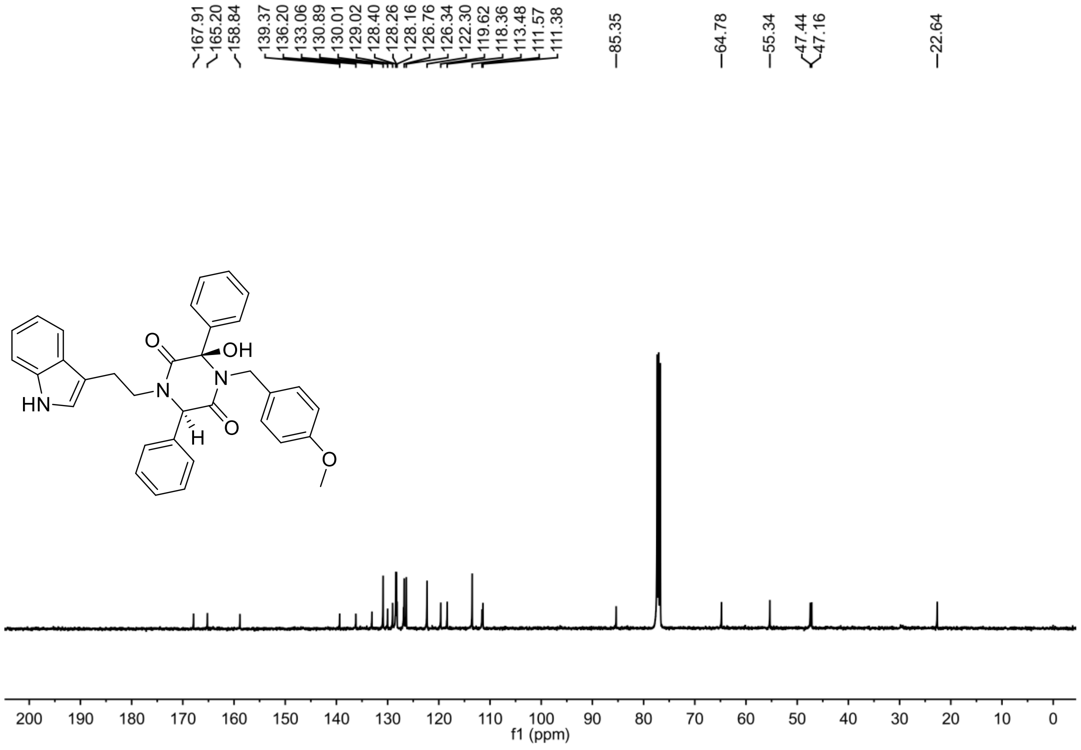

^{13}C NMR spectrum of **(±) 5n**

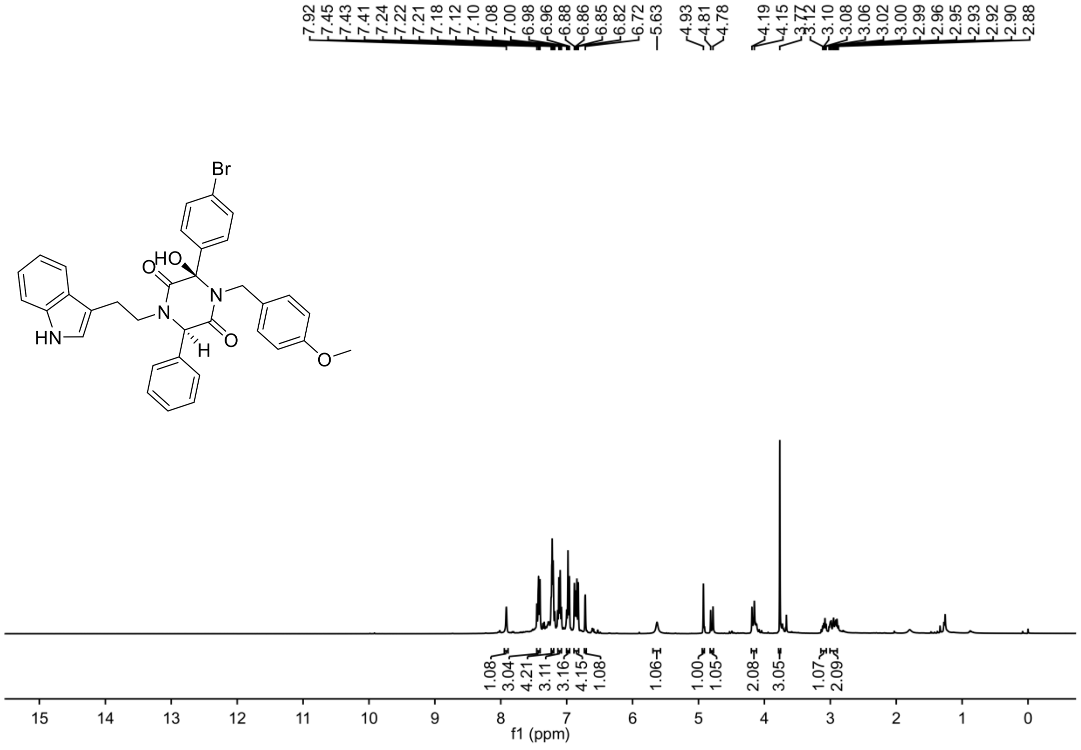

^{1}H NMR spectrum of **(±) 5o**

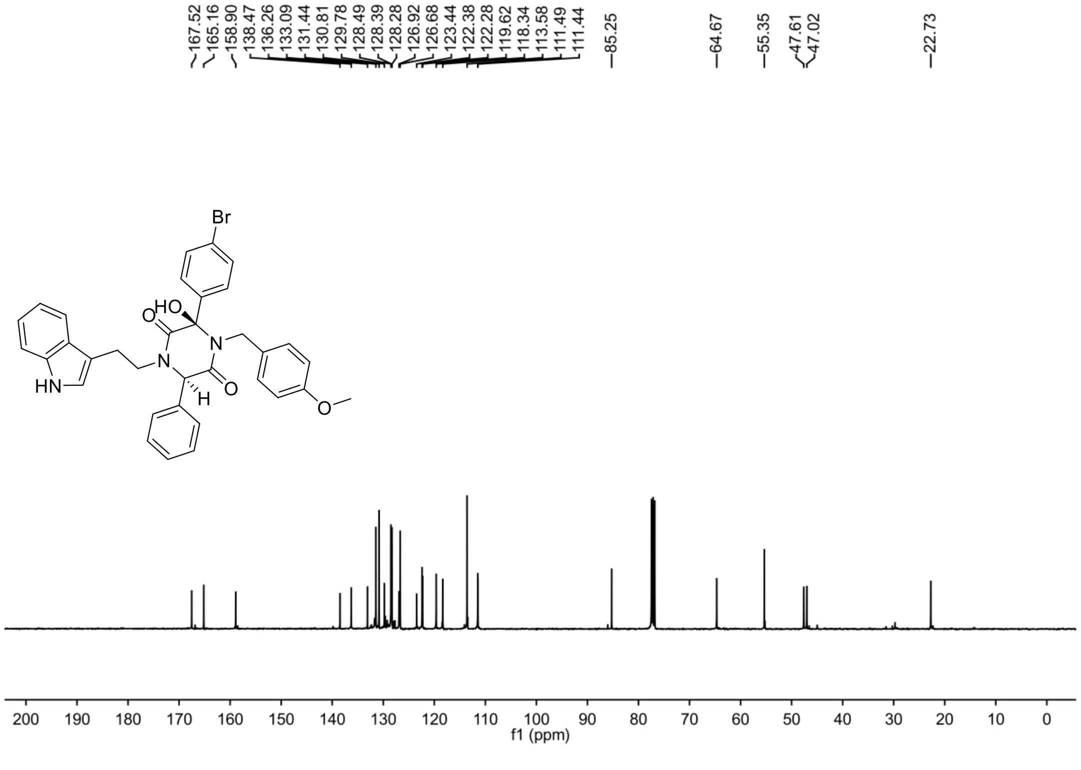

^{13}C NMR spectrum of **(±) 5o**

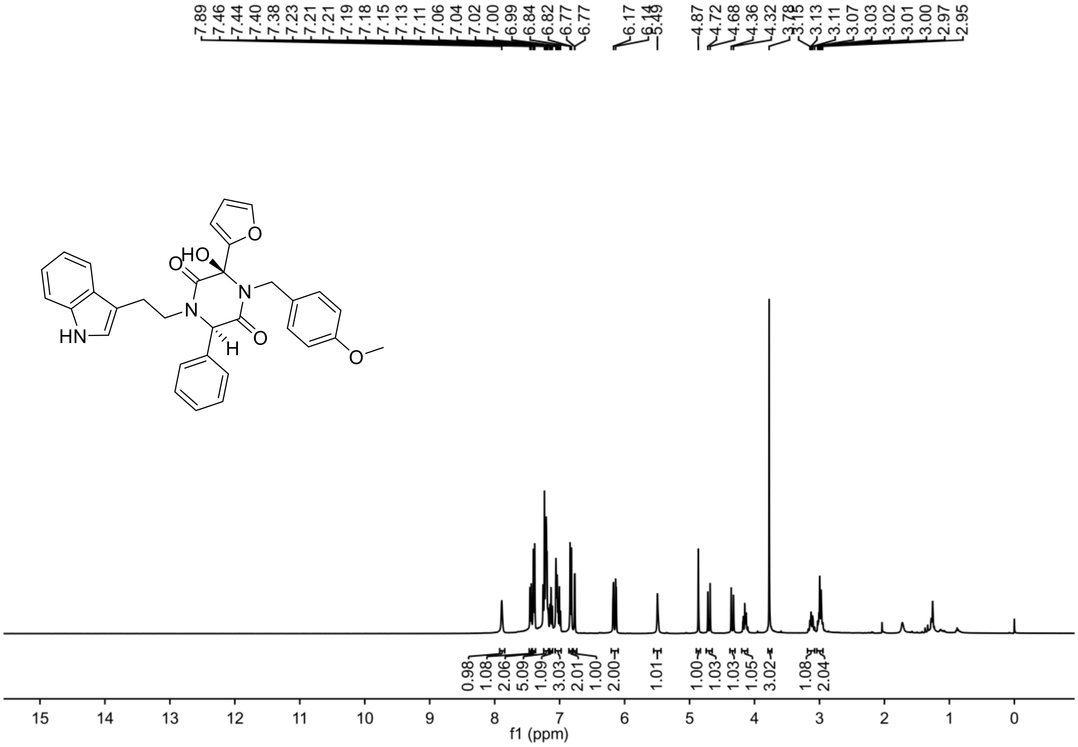

^{1}H NMR spectrum of **(±) 5p**

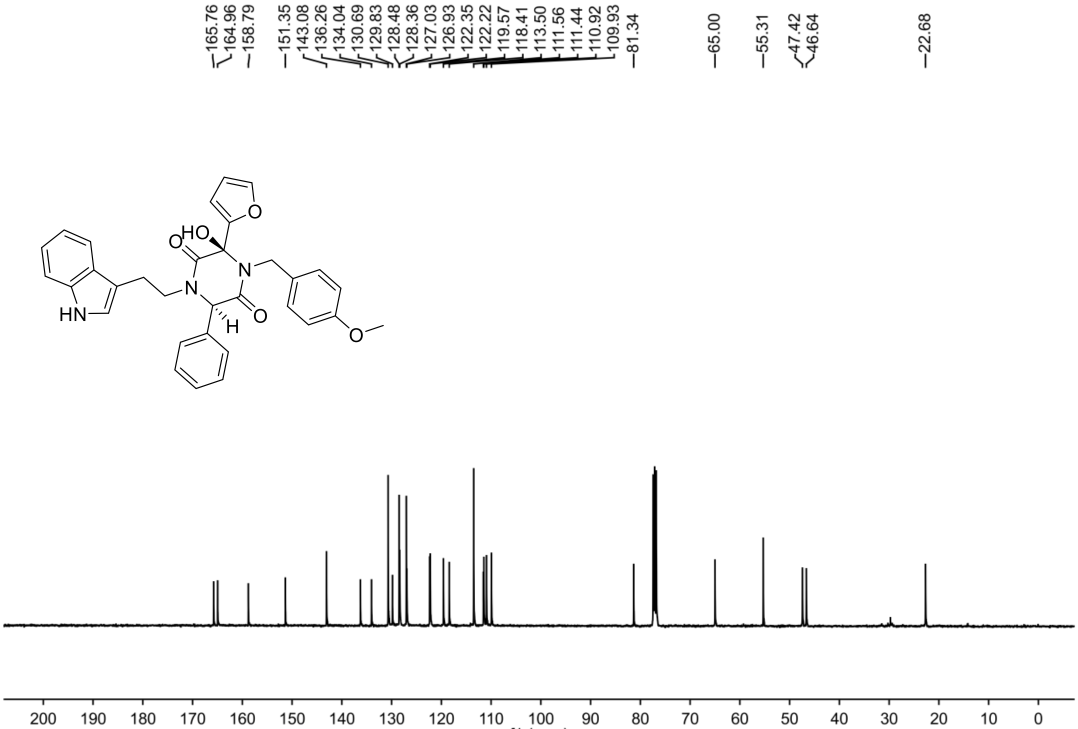

^{13}C NMR spectrum of **(±) 5p**
